# Supplementary material for: Sequence grammar underlying unfolding and phase separation of globular proteins
Source: Mol Cell. Author manuscript; Available in PMC 2024 Feb 6. (PMC10846692; doi:10.1016/j.molcel.2022.06.024)
Supplement: Supplementary Material [file EMS205330-supplement-Supplementary_Material.zip › 1-s2.0-S1097276522006074-mmc7.pdf]

Molecular Cell

Article

Sequence grammar underlying the unfolding and phase separation of globular proteins

Graphical abstract

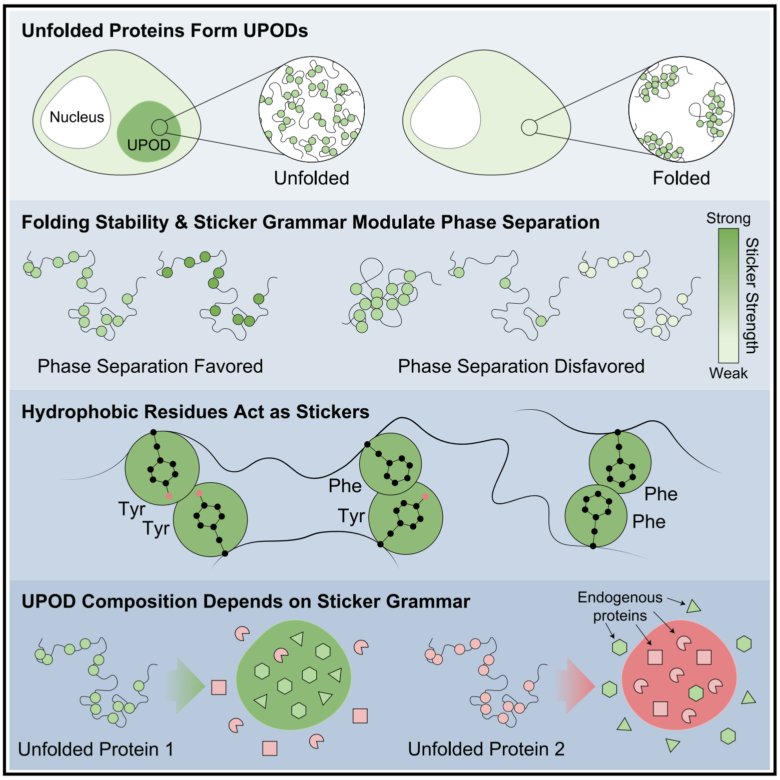

Highlights

- Unfolded states of globular proteins phase separate to form UPODs in cells
- Populations of unfolded molecules and the sticker grammar govern phase separation
- Hydrophobic residues act as stickers that engage in intermolecular interactions
- Sticker grammar also influences the gain-of-function recruitment into aberrant UPODs

Authors

Kiersten M. Ruff, Yoon Hee Choi, Dezerae Cox, ..., Sheena E. Radford, Rohit V. Pappu, Danny M. Hatters

Correspondence

pappu@wustl.edu (R.V.P.), dhatters@unimelb.edu.au (D.M.H.)

In brief

The phase separation of unfolded proteins is associated with the formation of unfolded protein deposits (UPODs). Ruff et al. show that for UPODs to form, the concentration of unfolded proteins must be above a threshold concentration and unfolded proteins must have a requisite number of cohesive motifs known as stickers.

Ruff et al., 2022, Molecular Cell *82*, 3193–3208
September 1, 2022 © 2022 The Authors. Published by Elsevier Inc.
https://doi.org/10.1016/j.molcel.2022.06.024

Molecular Cell

CellPress
OPEN ACCESS

Article

Sequence grammar underlying the unfolding and phase separation of globular proteins

Kiersten M. Ruff,[1,7] Yoon Hee Choi,[2,7] Dezerae Cox,[2] Angelique R. Ormsby,[2] Yoochan Myung,[3,4,5] David B. Ascher,[3,4,5] Sheena E. Radford,[6] Rohit V. Pappu,[1,*] and Danny M. Hatters[2,8,*]
[1]Department of Biomedical Engineering, Center for Science & Engineering of Living Systems, Washington University in St. Louis, St. Louis, MO 63130, USA
[2]Department of Biochemistry and Pharmacology and Bio21 Molecular Science and Biotechnology Institute, The University of Melbourne, Melbourne, VIC 3010, Australia
[3]Computational Biology and Clinical Informatics, Baker Heart and Diabetes Institute, Melbourne, VIC 3004, Australia
[4]Structural Biology and Bioinformatics, Department of Biochemistry and Pharmacology, The University of Melbourne, Melbourne, VIC 3010, Australia
[5]Systems and Computational Biology, Bio21 Institute, The University of Melbourne, Melbourne, VIC 3010, Australia
[6]Astbury Centre for Structural and Molecular Biology, School of Molecular and Cellular Biology, University of Leeds, Leeds LS2 9JT, UK
[7]These authors contributed equally
[8]Lead contact
*Correspondence: pappu@wustl.edu (R.V.P.), dhatters@unimelb.edu.au (D.M.H.)
https://doi.org/10.1016/j.molcel.2022.06.024

SUMMARY

Aberrant phase separation of globular proteins is associated with many diseases. Here, we use a model protein system to understand how the unfolded states of globular proteins drive phase separation and the formation of unfolded protein deposits (UPODs). We find that for UPODs to form, the concentrations of unfolded molecules must be above a threshold value. Additionally, unfolded molecules must possess appropriate sequence grammars to drive phase separation. While UPODs recruit molecular chaperones, their compositional profiles are also influenced by synergistic physicochemical interactions governed by the sequence grammars of unfolded proteins and cellular proteins. Overall, the driving forces for phase separation and the compositional profiles of UPODs are governed by the sequence grammars of unfolded proteins. Our studies highlight the need for uncovering the sequence grammars of unfolded proteins that drive UPOD formation and cause gain-of-function interactions whereby proteins are aberrantly recruited into UPODs.

INTRODUCTION

Protein homeostasis (proteostasis) is achieved by protein quality control machineries that regulate protein production, folding, trafficking, and degradation (Balch et al., 2008; Powers et al., 2009). A major function of the proteostasis machinery is to facilitate the correct folding of globular proteins that have a stable fold (Bobori et al., 2017; Reinle et al., 2022; Sontag et al., 2017). We refer to these proteins as intrinsically foldable proteins (IFPs). In cells, IFPs have a broad range of stabilities (Leuenberger et al., 2017) that depend on protein sequence and fold type (Figure 1A). IFPs can be classified as being unstable, U (bottom 10%); stable, S (top 10%); or of medium stability (remaining proteins). Analysis of the data of Leuenberger et al. shows that the sub-proteome comprising the least thermally stable IFPs (U) contain a higher fraction of disease-related proteins compared with the sub-proteome comprising the most stable IFPs (S) (Figure 1B). Decreased stability leads to a higher proclivity for sampling unfolded states under physiological conditions, leaving such proteins more susceptible to mutations that promote the formation of aberrant cellular deposits. Indeed, many IFPs with lower intrinsic folding stabilities have disease-associated mutations that promote concentration-dependent, aggregation-mediated phase separation and the formation of aberrant deposits (Figure 1C; Table S1) (Maier et al., 2009; Turner et al., 2005). For example, in the context of familial forms of amyotrophic lateral sclerosis (ALS), mutations to superoxide dismutase 1 (SOD1) affect the stability of the SOD1 dimer and promote the formation of protein deposits that accumulate through interactions among unfolded or partially unfolded monomeric states (Gomez and Germain, 2019; Meiering, 2008).

IFPs are defined by linked equilibria involving *folding* through intramolecular interactions, *binding* to components of the quality control machinery through specific heterotypic interactions, and *phase separation* through homotypic intermolecular interactions. The proposed triad of linked equilibria, inspired by findings from Frydman and coworkers (Kaganovich et al., 2008; Sontag et al., 2017), suggests that the formation of deposits through phase separation of misfolded, partially unfolded, or unfolded proteins,

This is an open access article under the CC BY-NC-ND license (http://creativecommons.org/licenses/by-nc-nd/4.0/).

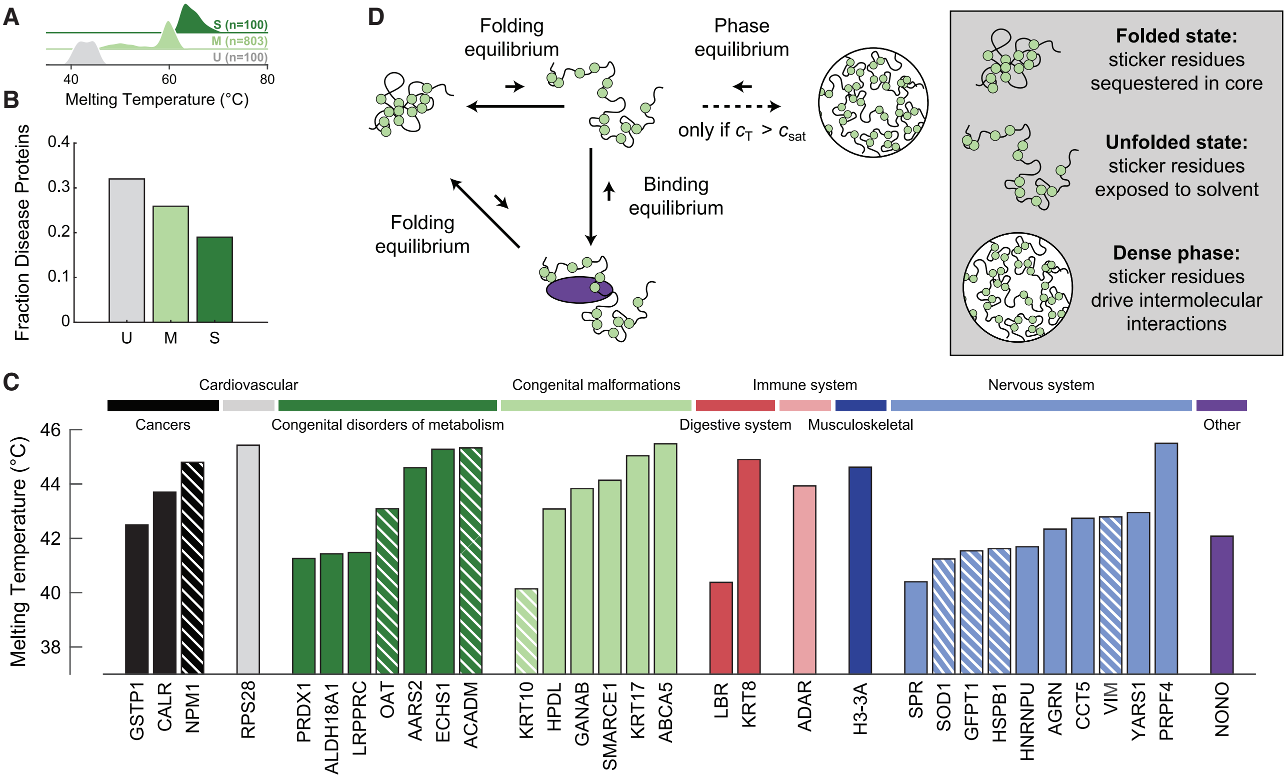

Figure 1. Normal cellular function requires balancing an interconnected equilibria triad of folding, binding to components of the quality control machinery, and phase separation

(A) Probability density estimates of the melting temperatures for human proteins in the unstable (U), medium stable (M), and stable (S) classes as defined by Leuenberger et al. and extracted from ProThermDB (Table S1) (Leuenberger et al., 2017; Nikam et al., 2021). In accordance with Leuenberger et al., we classified the bottom 10% of proteins in terms of melting temperature as unstable, the top 10% as stable, and those remaining as medium stable.

(B) The fraction of proteins in the U, M, and S classes that are associated with Kyoto Encyclopedia of Genes and Genomes (KEGG) disease proteins (Table S1) (Kanehisa and Goto, 2000).

(C) Melting temperatures of the 32 unstable human proteins associated with disease. Proteins are grouped by KEGG disease type. Stripes in the bars indicate that there is experimental evidence for disease-associated mutations leading to aggregation-mediated phase separation (Table S1).

(D) Schematic of the interconnected equilibria of folding, binding with protein quality control machinery, and phase separation. The green circles denote stickers, and the purple oval denotes chaperone binding. Here, c_{tot} denotes the total IFP concentration. When $c_{tot} > c_{sat}$, the homogeneous well-mixed phase is saturated, and the system separates into two coexisting phases.

See also Figure S1 and Table S1.

driven by homotypic interactions, might be part of the normal processing of unfolded proteins (Figure 1D).

Our goal was to uncover the principles that govern phase separation driven by homotypic interactions among unfolded proteins. Under folding conditions, IFPs can sample folded and unfolded states, where the latter are distinct from states accessed in the presence of high concentrations of denaturants (Peran et al., 2019). The folding-unfolding equilibrium is also regulated by binding to components of the quality control machinery (Powers et al., 2009). For instance, chaperones bind to exposed hydrophobic patches of amino acids in unfolded states of IFPs to mediate the folding process or deliver IFPs for degradation.

IFPs are also characterized by a phase equilibrium whereby they undergo concentration-dependent phase separation. These transitions are driven by homotypic interactions among unfolded molecules whereby, in its simplest form, a protein plus solvent system separates into a dilute, protein-deficient phase and a coexisting dense, protein-rich phase (Mathieu et al., 2020; Pappu et al., 2008; Posey et al., 2018a). Phase separation, which results from a combination of specific- and non-specific homotypic interactions, is a density transition, referred to as aggregation-mediated phase separation (Posey et al., 2018b). For a given set of solution conditions, the strengths of driving forces for phase separation driven by homotypic interactions can be quantified by a saturation concentration, c_{sat}, which is the threshold concentration of the protein above which it separates into coexisting dilute and dense phases (Figure S1A) (Wang et al., 2018). Thus, when the total concentration of protein is above c_{sat}, a phase equilibrium exists between molecules in the dilute phase and molecules in the dense phase.

Although recent work has co-opted the term phase separation to refer to the coexistence of two liquid phases, the formal definition of phase separation does not impose any constraints on

the material properties of coexisting phases. Indeed, the use of saturation concentrations to quantify driving forces for forming protein-rich deposits via aggregation-mediated phase separation predates the current focus on liquid-liquid phase separation (LLPS) alone (Ciryam et al., 2017; Ciryam et al., 2013; Crick et al., 2006; Crick et al., 2013; Garai et al., 2008; Pappu et al., 2008). Phase separation can give rise to an assortment of coexisting phases, and the appropriate prefix, such as liquid-liquid, liquid-solid, solid-solid, etc., depends on the material properties of the coexisting phases (Figure S1B).

For many IFPs, aberrant phase separation appears to be the result of concentration-dependent interactions among unfolded proteins (Balchin et al., 2020; Clark, 2004; Hartl, 2016; Vendruscolo and Dobson, 2005; Ryno et al., 2013; Solomon et al., 2012; Song, 2018). For example, soluble wild-type (WT) SOD1 exists as a homodimer, stabilized by metal binding and an intra-subunit disulfide bond. Aberrant phase separation and formation of SOD1 deposits is driven by interactions among unfolded states of SOD1 (Nordlund et al., 2009). These results suggest several testable hypotheses. First, disease-related mutations likely restructure the triad of equilibria by increasing the concentration of unfolded molecules and thus decreasing the total protein concentration required to drive phase separation. Second, because phase separation is driven primarily by interactions among unfolded molecules, the cohesive motifs (stickers) (Choi et al., 2020) that drive phase separation must be accessible to drive homotypic interactions among unfolded molecules. Third, components of the protein quality control machinery can bind unfolded molecules and thereby weaken their ability to engage in homotypic interactions that lead to phase separation.

Here, we focused on answering the following questions: are all unfolded states of IFPs equivalent as drivers of phase separation and the formation of aberrant, *de novo* unfolded protein deposits (UPODs) in cells, or must the unfolded states expose distinctive stickers that can drive phase separation? Can chaperones destabilize the formation of aberrant UPODs? Are all UPODs compositionally equivalent, or do different compositions of stickers recruit different proteins based on the physiochemical properties of the stickers? We answered these questions by utilizing the model protein barnase, a monomeric globular protein whose structure, stability, and folding *in vitro* (Dalby et al., 1998; Matthews and Fersht, 1995) and *in vivo* (Wood et al., 2018) have been studied extensively.

Barnase is a bacterial ribonuclease. The catalytically inert H102A variant (referred to as the WT here) is benign in mammalian cells (Wood et al., 2018). The population of molecules in folded versus unfolded states is dictated by the free energy of unfolding: $\Delta G^{\circ}_{U} = (G^{\circ}_{U} - G^{\circ}_{F})$. Here, G°_{U} and G°_{F} are the standard state free energies of the unfolded and folded states, respectively. The relative fraction of molecules in unfolded versus folded states increases monotonically as ΔG°_{U} decreases in favor of the unfolded state. For proteins with large positive values of ΔG°_{U}, essentially ~100% of the molecules will be folded. Conversely, for proteins with large negative values of ΔG°_{U}, essentially ~100% of the molecules will be unfolded.

WT barnase, fused to mTFP1 at the N terminus and Venus at the C terminus, does not form deposits in mammalian cells (Wood et al., 2018). In contrast, variants for which ΔG°_{U} becomes less positive or even negative will have diminished stability. Increased access to unfolded states, through decreased stability, increases the concentration of unfolded proteins, leading to the formation of deposits in mammalian cells. Unfolded states of barnase are also known to engage with components of the quality control machinery (Wood et al., 2018). Together, these results imply that we can use barnase to interrogate the three-way interplay of protein stability, phase separation, and engagement with the quality control machinery.

RESULTS

Phase separation is driven by the interactions among unfolded barnase molecules

We deployed an optoDroplet system to uncover the sequence grammar that underlies the phase separation of mutational variants of barnase molecules in live cells. The optoDroplet system was developed to study the phase separation of multivalent proteins using a precise and controllable reaction triggered by blue light (Shin et al., 2017). The system involves a fusion of the protein of interest to the photoactivatable Cry2 domain (Hsu et al., 1996; Lin et al., 1998) and a fluorescent protein reporter (Figure 2A). Although Cry2 forms sub-microscopic oligomers upon blue-light illumination, it does not drive phase separation, even upon light activation (Lin et al., 1998). When Cry2 is fused to a domain that can undergo phase separation, the oligomerization of Cry2 reduces c_{sat} for phase separation. Hence, this fusion design allows the quantitative and inducible comparison of apparent and relative c_{sat} values for protein sequences that are fused to Cry2 (Shin et al., 2017).

The intrinsically disordered region (IDR) of DDX4 was used as a positive control (Figure 2B) of the optoDroplet setup (Brady et al., 2017; Nott et al., 2015; Shin et al., 2017). In contrast to the IDR of DDX4, WT barnase did not undergo phase separation when Cry2 was light activated (Figure 2B). However, the (I25A, I96G) double mutant (referred to as Ex4) has a finite probability of accessing unfolded states under physiological conditions, and it undergoes phase separation in a blue-light-dependent manner (Figures 2B and 2C). Therefore, phase separation, driven by interactions among unfolded barnase molecules, can be assessed in a controlled manner without the confounding effects of slow kinetics that characterize the formation of UPODs in cells.

A combination of protein destabilization and a distinct sequence grammar is required for UPOD formation

We examined different mutants of barnase to titrate the impact of ΔG°_{U} on phase separation. ΔG°_{U} for these variants ranged from +18.7 kJ/mol (highly stable) to −0.8 kJ/mol (highly unstable) (Wood et al., 2018). Variants with ΔG°_{U} values above +13.0 kJ/mol were resistant to phase separation, whereas those with ΔG°_{U} values below this threshold underwent phase separation (Figure 3A). If the abundance of unfolded proteins, dictated by ΔG°_{U}, is the sole determinant of the driving forces for phase separation, then there should be a threshold concentration of unfolded proteins, c^*, above which the system separates into dilute and dense phases (Figure 3B). This concentration quantifies the saturation threshold of the unfolded species. The value

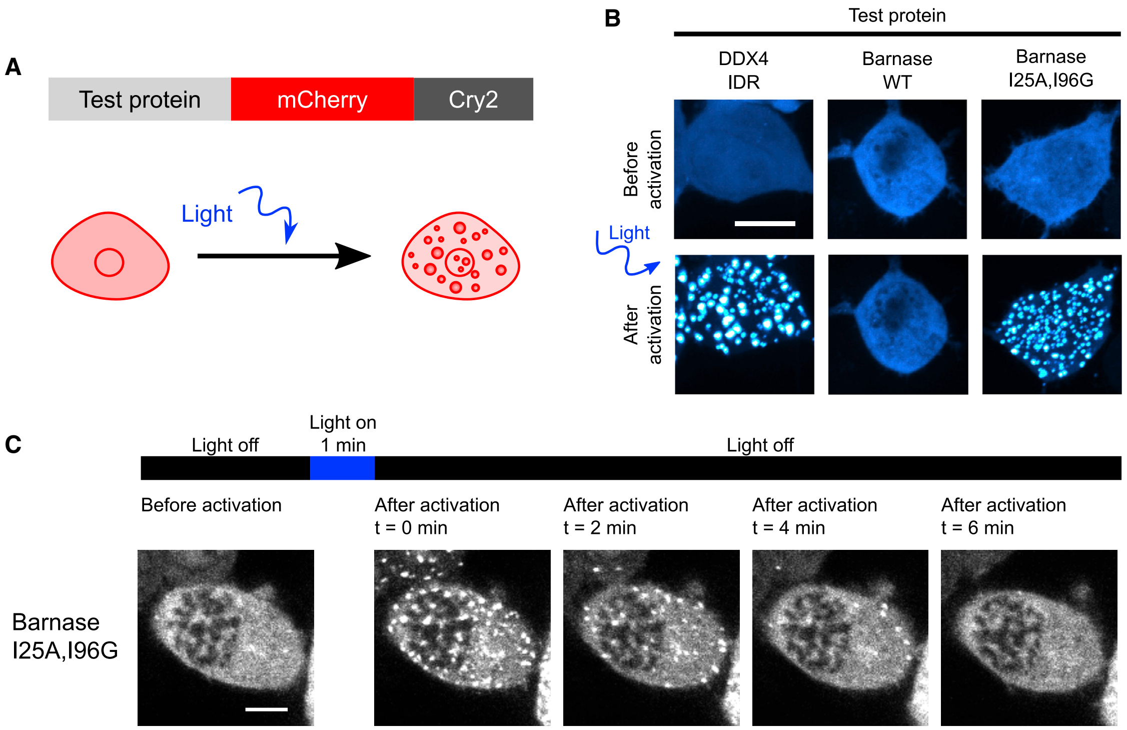

Figure 2. Phase separation is driven by interactions among unfolded barnase molecules

(A) Schematic of constructs used for the optoDroplet assay.

(B) Representative confocal micrograph images of Neuro2a cells transfected with DDX4 IDR (positive control), WT barnase, and the destabilizing barnase variant (I25A, I96G) optoDroplet constructs before and after light activation.

(C) Time-lapsed confocal imaging of live Neuro2a cells expressing the I25A, I96G barnase-optoDroplet construct. Scale bars in panels B and C correspond to 10 μm.

of c^*, which is defined as $c^* = p_U \times c_{sat}$, is a product of the apparent saturation concentration, c_{sat}, comprising folded and unfolded barnase molecules, and p_U, which is the fraction of molecules in the unfolded state. If phase separation is driven exclusively by concentrations of unfolded proteins, then variants with the lowest fraction of unfolded proteins will have the highest c_{sat} values because $c_{sat} = c^* \times (p_U)^{-1}$.

Previous studies have shown that many IFPs tend to have lower stabilities in cells than would be predicted based on estimates of ΔG°_U from *in vitro* measurements (Danielsson et al., 2015; Gnutt et al., 2019; Wood et al., 2018). Also, the appendages, Cry2 and mCherry, are likely to alter the ΔG°_U values when compared with estimates from *in vitro* measurements with untagged barnase molecules in dilute solutions. Accordingly, our estimates of c^* use a constant offset for ΔG°_U vis-à-vis values measured *in vitro* (see STAR Methods). For each barnase variant, we estimated c_{sat} using the dilute phase fluorescence intensity before and after light activation (Figures S2A–S2C). The lowest dilute phase fluorescence intensity at which we observe divergent behavior before and after light activation is the threshold concentration for the appearance of droplets (STAR Methods). Measured c_{sat} values are best described by a model where $c^* \approx 10.83$ fluorescence intensity units (a.u.) that uses an offset of −12.9 kJ/mol (−3.1 kcal/mol) for all ΔG°_U values (Figure S2C). The analysis summarized in Figure 3C shows that the barnase variants falls into three categories. First, phase separation was not observed when the concentration of unfolded molecules was too low. Second, phase separation was observed for $p_U \approx 0.5$ and the c_{sat} was accurately predicted by c^*. Third, as p_U approached one, c_{sat} was no longer accurately predicted by c^*. Instead, the underlying sequence grammar, namely the intrinsic stickiness of the molecule, dictates the driving force for phase separation (Lang et al., 2015).

Mutations that destabilize the folded states of IFPs often do so by weakening the hydrophobic core. Accordingly, if the residues that drive chain collapse and phase separation are equivalent (Bremer et al., 2022; Martin et al., 2020; Zeng et al., 2020), then destabilizing mutations would be expected to weaken the driving forces for phase separation of unfolded proteins. Therefore, we proposed that even if all protein molecules were unfolded ($p_U \approx 1$), phase separation would only occur if the requisite sticker residues were present and accessible. To test this hypothesis, we used the CamSol method (Sormanni et al., 2015) to calculate relative solubilities normalized to that of WT barnase. We found that all the mutational variants were predicted to be more soluble than WT. Furthermore, three of the four barnase variants that showed a higher measured c_{sat} compared with the predicted c_{sat} strongly increased the solubility compared with the WT barnase (Figure 3C). These results

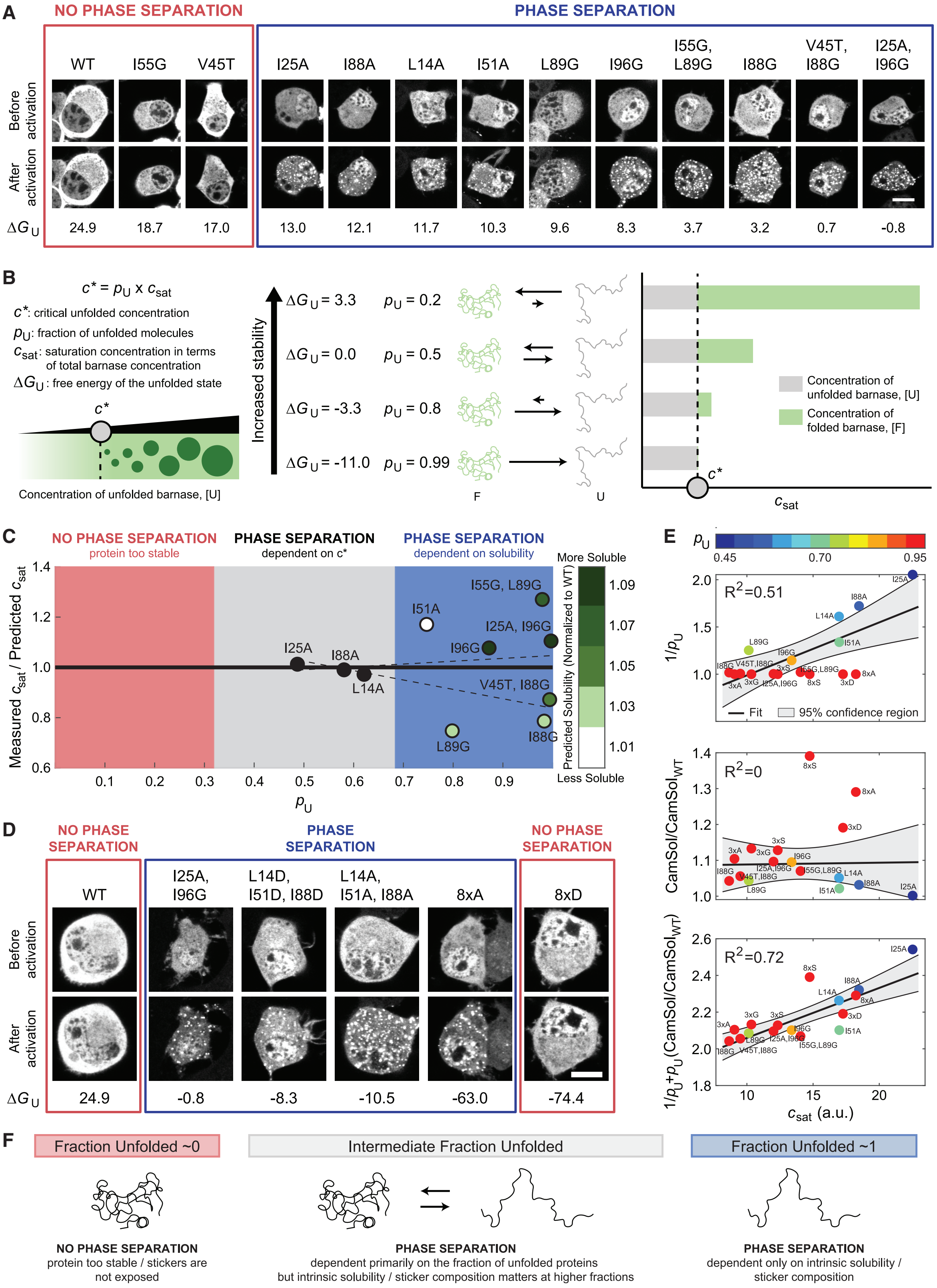

(legend on next page)

suggested the following takeaways. When IFPs become primarily unfolded, the intrinsic solubility of the protein dictates the c_{sat}. The CamSol predictions suggest that the interactions among the hydrophobic residues of unfolded molecules are important for driving the phase separation of IFPs.

To explore the importance of the requisite number of stickers for interactions among unfolded proteins, we introduced additional mutations into barnase that effectively ablated the folded state ($\Delta G^{\circ}_{U} \leq -10$ kJ/mol). This enabled quantification of driving forces for phase separation based solely on the properties of unfolded states (Figures 3D, 3E, and S2D). The mutations were chosen to alter the chemical environment of bulky hydrophobic residues that would normally be in the folded core. This includes triple mutations L14X, I51X, and I88X, with X being A, G, S, or D. These mutations are referred to as the 3×X variants. We also introduced octuple mutations L14X, L42X, I51X, L63X, I76X, I88X, L89X, and I96X, with X as A, S, or D, referred to as 8×X variants. In terms of hydrophobicity, the substitutions should follow the trend A > G > S > D (Kyte and Doolittle, 1982).

All variants except 8×D showed intracellular phase separation in the concentration regimes that we explored (Figures 3D, 3E, and S2D). However, even though all variants were predicted to have a c_{sat} of ~11 a.u. based on their p_U, the measured c_{sat} values spanned a range from ~9 to ~18 a.u. (Figures 3E and S2D). These results suggest that not all unfolded states are equivalent as drivers of phase separation. Instead, sequence-specific stickers modulate the driving force for phase separation. Combining data for all the barnase variants, we found that neither p_U nor the intrinsic solubility of the unfolded state alone were suitable predictors of the measured c_{sat} values (Figure 3E; $R^2 = 0.51$ and 0, respectively). However, consideration of both p_U and the intrinsic solubility of each variant improves correlation with the measured c_{sat} values (Figure 3E; $R^2 = 0.72$). This suggested that the c_{sat} of IFPs with intermediate values of p_U are dictated primarily by p_U, whereas the c_{sat} values of IFPs with p_U~1 should be dictated primarily by intrinsic solubilities of unfolded proteins (Figure 3F). Overall, these results suggest that phase separation requires that the unfolded state be favorably populated *and* that sticker-mediated interactions among unfolded molecules be minimally disrupted by mutations that destabilize the folded state.

Phe and Tyr function as stickers that drive the phase separation of unfolded barnase

To identify specific residues that function as stickers, we performed atomistic simulations of unfolded states of WT barnase. Residues predicted to be optimal stickers should have a higher probability of being in contact with other residues in the unfolded ensembles (Martin et al., 2020). We found that hydrophobic residues have the highest mean contact probability (Figure 4A). Of particular interest is the identification of Tyr and Phe, given their roles as stickers that drive the phase separation of intrinsically disordered prion-like low-complexity domains (Bremer et al., 2022; Lin et al., 2017; Martin et al., 2020; Wang et al., 2018) and in forming the selectivity filter of nuclear pore complexes (Frey et al., 2006). Accordingly, we tested the importance of aromatic residues as stickers for driving UPOD formation.

To avoid confounding factors arising from the folded state, we introduced mutations into the 8×A variant, which is completely unfolded but still drives phase separation (Figures 3D and 3E). Three categories of mutations were examined (Figure 4B). First was the replacement of aromatic residues with Ser. This should reduce the number of stickers (Bremer et al., 2022). Second was the replacement of Phe with Tyr. This should increase the sticker strength (Bremer et al., 2022). Third was the replacement of polar residues with Tyr. This should increase the number of stickers (Bremer et al., 2022; Martin et al., 2020). The effects of these mutations were assessed using the optoDroplet assay (Figures 4C, 4D, and S3). Decreasing the number of stickers weakened the driving forces for phase separation, and mutations replacing polar residues with Tyr enhanced the driving forces for phase separation. Substituting one or more Phe residues with Tyr had minimal impact on c_{sat}. This suggests that Phe and Tyr have equivalent efficacy as stickers when phase separation is driven by interactions among unfolded barnase molecules.

Interactions that drive the phase separation of unfolded states have an equivalent impact on deposit formation

If phase separation is a generic density transition, then the apparent c_{sat} values extracted using the optoDroplet assay should be equivalent to threshold concentrations extracted using an orthogonal assay that probes the formation of protein deposits in cells. We examined deposit formation using an assay

Figure 3. A combination of protein destabilization and a distinct sequence grammar are required for UPOD formation

(A) Representative confocal micrograph images of Neuro2a cells transfected with the barnase-optoDroplet constructs as shown before and after light activation. Red box indicates constructs that do not undergo phase separation, whereas the blue box denotes constructs that do. Scale bars correspond to 10 μm. ΔG°_{U} values are given in kJ/mol.

(B) Model for how c_{sat} should change if stability, and thus a critical concentration of unfolded proteins, c^*, is all that matters for phase separation.

(C) Measured versus predicted c_{sat} as a function of p_U (Figures S2B and S2C; Table S2) . The predicted c_{sat} values were determined by globally fitting all barnase variants to $c_{sat} = c^*/p_U$ for a constant c^* and an offset in ΔG°_{U} (STAR Methods). The best fit was for $c^* = 10.83$ a.u. and $\Delta G^{\circ}_{U} = -12.9$ kJ/mol. Variants are colored by their normalized CamSol solubility score. Dashed lines denote the fitted confidence interval determined by 1,000 bootstrapped trials of the variants, where the variants were picked based on the degree to which they modulated the hydrophobic and hydrophilic blobs from WT (STAR Methods).

(D) Representative confocal micrograph images of Neuro2a cells transfected with the additional barnase-optoDroplet constructs with negative ΔG°_{U} values. Scale bars indicate 10 μm. ΔG°_{U} values are given in kJ/mol.

(E) Comparison of c_{sat} and only stability ($1/p_U$), only predicted solubility ($CamSol/CamSol_{WT}$), or a combination of stability and solubility ($1/p_U + p_U(CamSol/CamSol_{WT})$) using linear regression. Barnase variants are colored by their expected p_U given the offset in ΔG°_{U} of −12.9 kJ/mol. The 8×S variant was treated as an outlier in these analyses, given the lack of cellular data at intermediate concentrations. Thus, the accuracy of the extracted c_{sat} was not clear (Figure S2D, gray box).

(F) Summary of what features drive the phase separation of IFPs.

See also Figure S2 and Table S2.

CellPress
OPEN ACCESS

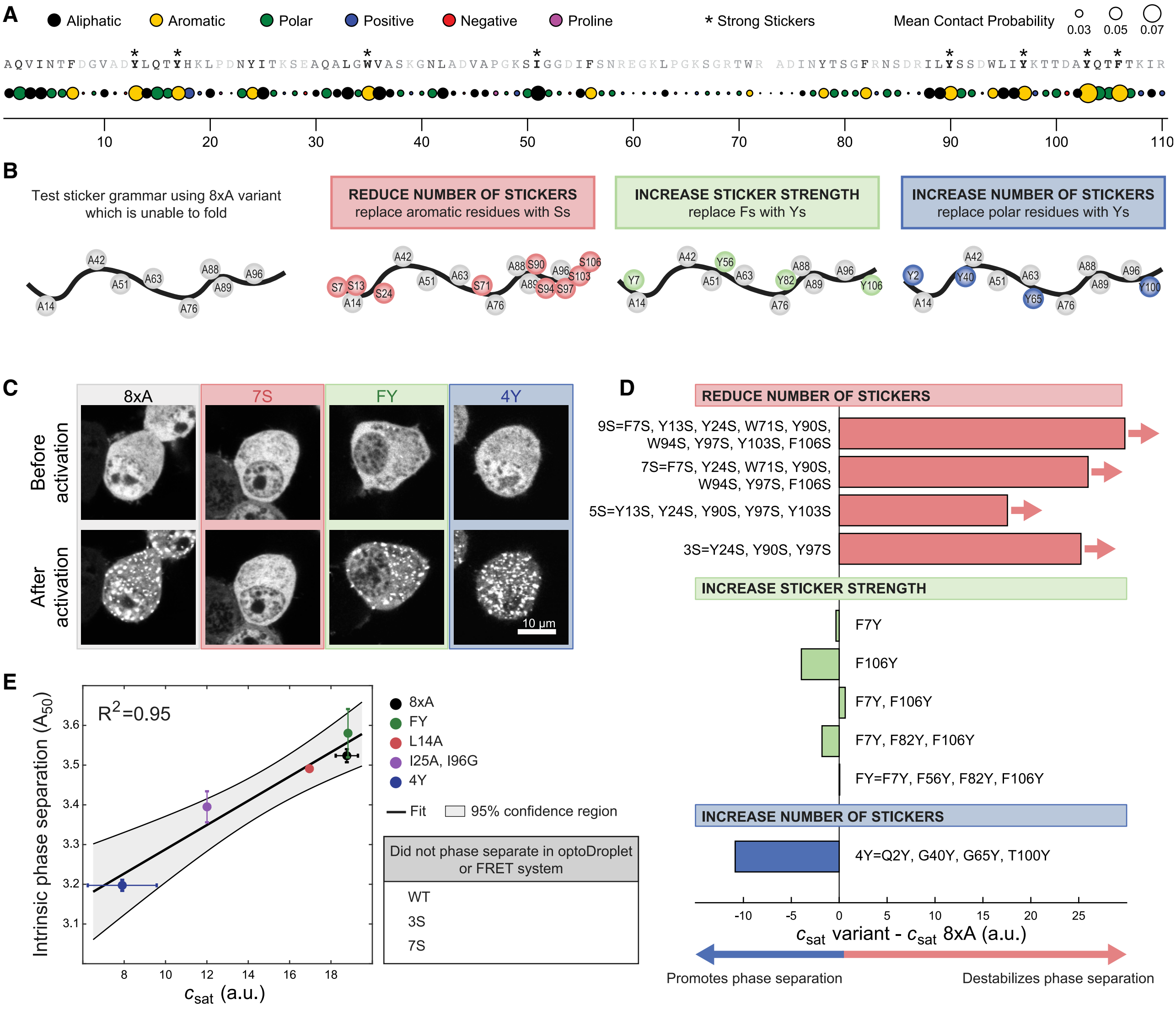

Figure 4. Phe and Tyr function as stickers that drive the phase separation of unfolded barnase

(A) Mean contact probability for each residue quantified from the atomistic simulations of the unfolded states of WT barnase. The WT sequence is listed across the top, and each residue is shaded based on its mean contact probability. Residues highlighted by * denote strong stickers. Strong stickers are those residues that have a mean contact order greater than the maximum mean contact order from a Flory random coil simulation (STAR Methods).

(B) Schematic of constructs used to test sticker grammar. In all cases, the base construct was 8×A, which has eight hydrophobic residues mutated to A (gray circles). Additional mutations used to test sticker grammar are shown in colored circles. Letters denote the residue the position is mutated to.

(C) Representative confocal micrograph images of Neuro2a cells transfected with the sticker barnase-optoDroplet variant constructs.

(D) Comparison of the c_{sat} values of each sticker barnase-optoDroplet variant construct with the c_{sat} of the 8×A construct in arbitrary units. Bars with arrows indicate that a c_{sat} value could not be extracted for these constructs and must be at least above the value of the bar.

(E) Comparison of the intrinsic phase separation of barnase as fusions to fluorescent proteins mTFP1 and Venus, using the A_{50} analysis, to c_{sat} values of barnase in the optoDroplet format (R^2 = 0.95 for linear regression). Error bars indicate standard deviations.

Also, see Figure S3 and Table S3.

involving fluorescence resonance energy transfer (FRET). FRET was measured with mTFP1 (donor) and Venus (acceptor) fluorescent proteins fused to the barnase constructs, where acceptor (Venus) fluorescence versus donor fluorescence (mTFP1) provides a readout on the assembly of barnase molecules (Wood et al., 2018). We derived estimates of the concentration of barnase in cells at which 50% of the cells contain deposits (A_{50} value). Lower concentrations correspond to stronger driving forces for deposit formation. We found a strong positive correlation between the A_{50} and c_{sat} values (R^2 = 0.95 for linear regression) (Figure 4E). Variants that did not form deposits also did not form droplets. These measurements demonstrate the equivalence of driving forces for deposit formation and droplet formation in the optoDroplet assay.

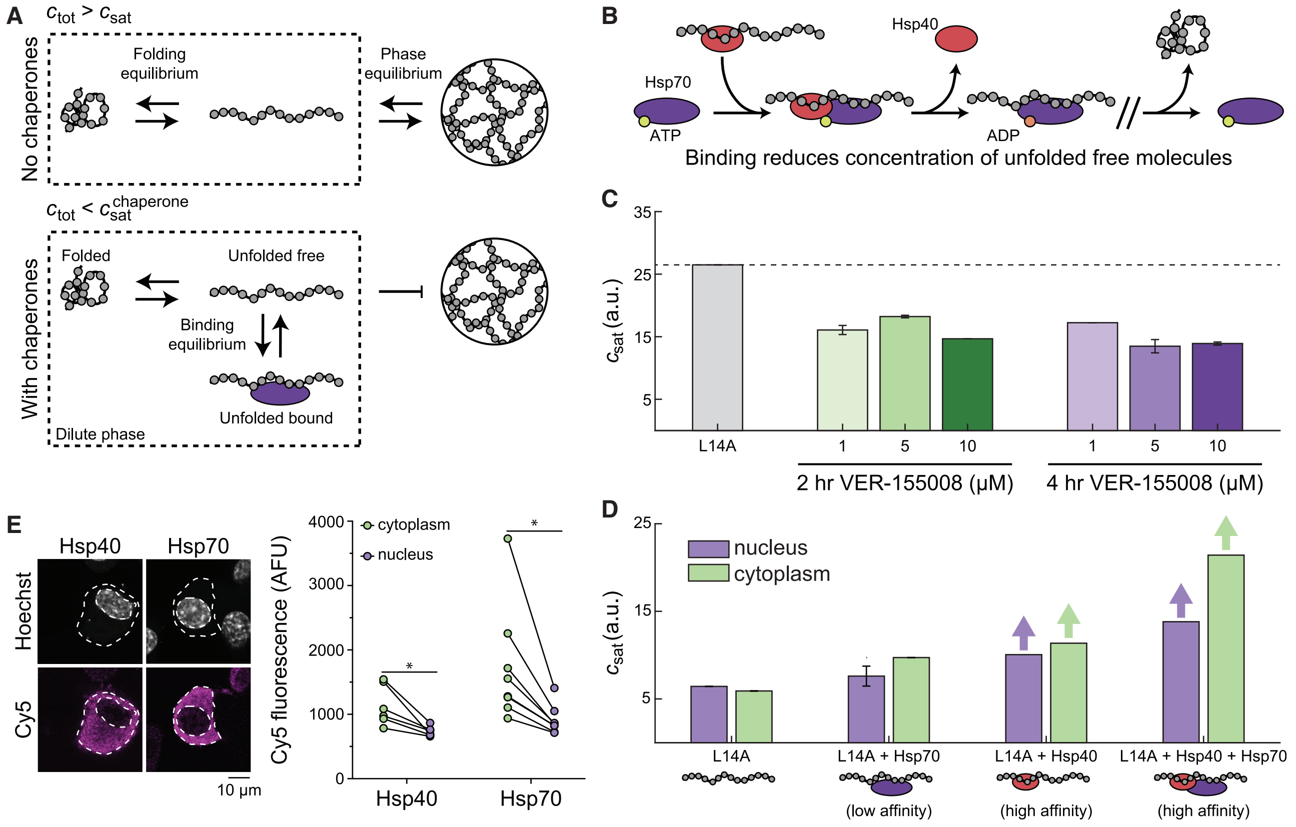

Figure 5. Molecular chaperones suppress phase separation

(A) In the absence of chaperones, the dilute phase consists of folded and unfolded barnase. There exists a phase equilibrium when the total concentration of barnase, c_{tot}, is greater than c_{sat}. In the presence of chaperones, barnase in the dilute phase consists of three dominant states: folded, unfolded free, and unfolded bound to chaperones. At the same total concentration of barnase as in the absence of chaperones, barnase cannot phase separate because c_{tot} is less than the saturation concentration needed in the presence of chaperones, $c_{sat}^{chaperone}$, given that chaperone binding reduces the concentration of free unfolded barnase.

(B) Basic model for chaperone function. Hsp40 binds the unfolded molecule and forms a ternary complex with Hsp70 in the ATP-bound state. ATP hydrolysis leads to the release of Hsp40 and the formation of a high-affinity complex between Hsp70 and the unfolded substrates.

(C) c_{sat} for the L14A barnase-optoDroplet variant construct transiently transfected in Neuro2a cells in the absence or presence of different dosages of the Hsp70 inhibitor VER-155008. Dashed line corresponds to the c_{sat} of L14A in the absence of the inhibitor. Error bars denote the standard deviation from 50 bootstrapped trials.

(D) c_{sat} for the L14A barnase-optoDroplet variant construct in the absence or presence of overexpressed chaperones. Bars with arrows indicate that a c_{sat} value could not be extracted for these systems and must be at least above the value of the bar. Error bars denote the standard deviation from 50 bootstrapped trials.

(E) Confocal images of Neuro2a cells transfected with V5-tagged DNAJB1 (Hsp40) or HSPA1A (Hsp70). Cells were stained by immunofluorescence for the V5-tag (Cy5), and the nucleus was stained with Hoechst 33342. Graphs show the quantitation of immunofluorescence. Data shown as paired samples from individual cells. Paired t test results shown; * $p < 0.05$.

See also Figure S4 and Table S4.

Molecular chaperones suppress the phase separation of unfolded barnase molecules

Next, we explored how chaperones influence phase separation driven by interactions among unfolded barnase molecules. Components of the chaperone system can bind to unfolded barnase either in the dense (Figure S4) or dilute phase (Wood et al., 2018). If binding to unfolded proteins in the dilute phase is stronger than in the dense phase, then c_{sat} in the presence of the chaperone, designated as $c_{sat}^{chaperone}$, will be greater than c_{sat} in the absence of the chaperone. Conversely, if binding to unfolded proteins in the dilute phase is weaker than in the dense phase, then $c_{sat}^{chaperone}$ will be lower than c_{sat} in the absence of the chaperone. Preferential binding, which would be true of chaperones that function independently of ATP hydrolysis, is referred to as polyphasic linkage (Ruff et al., 2021b; Wyman and Gill, 1980). In the dilute phase, there should be three states of barnase, namely folded barnase, unfolded barnase, and unfolded barnase bound to chaperones (Figure 5A). Members of the Hsp70 and Hsp40 families can bind to barnase in the dilute phase and suppress UPOD formation (Wood et al., 2018). Therefore, we proposed that while the total concentration of unfolded proteins (free + bound) would be higher in the presence of chaperones, the fraction of molecules capable of phase separation should be lowered (Figure 5A).

The canonical model is that Hsp40 binds substrates and then forms a ternary complex with Hsp70 in the ATP-bound state

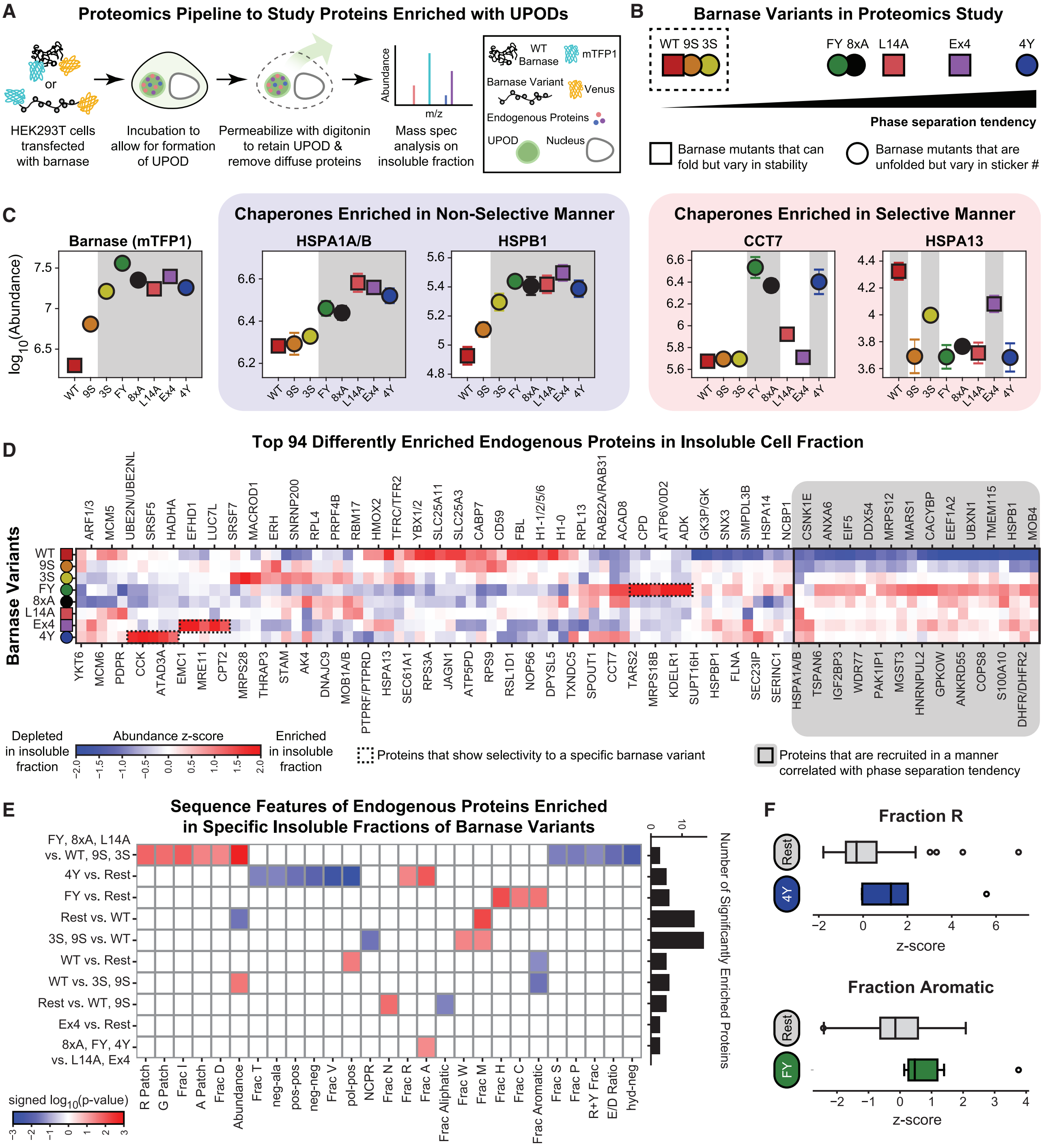

Figure 6. UPODs sequester and enrich cellular proteins through interactions governed by physical chemistry

(A) Schematic of the proteomics workflow to extract the compositional profiles of insoluble fractions of cells enriched with specific barnase variant UPODs.

(B) Barnase variants used for the proteomics study vary in phase separation tendency (c_{sat}), stability (ΔG°_{U}), and sticker composition. Variants within the dashed box were not found to phase separate at the concentrations tested.

(C) Abundance of barnase (mTFP1) and four representative chaperones in barnase-specific insoluble fractions. Barnase variants are sorted based on their phase separation tendency. Selectivity refers to recruitment not correlated with the phase separation tendency of the barnase variants. Shaded gray regions denote the UPODs that the given protein is significantly enriched in, as determined by the Fisher's LSD test following an ANOVA test. Error bars denote the standard error of the mean of four replicates.

(legend continued on next page)

(Alderson et al., 2016; Jiang et al., 2019) (Figure 5B). ATP hydrolysis correlates with the release of Hsp40 and the formation of a high-affinity complex between the substrate and Hsp70. If unfolded barnase molecules are a target of the Hsp40/Hsp70 system, we expected that inhibiting Hsp70 would promote phase separation, decreasing the c_{sat} of barnase molecules. Indeed, the treatment of the cells with the Hsp70-specific inhibitor compound VER-155008 (IC_{50} = 0.5 μM) caused a lowering of the optoDroplet estimated c_{sat} of variant L14A (Figure 5C).

To further assess the impact of chaperones on the phase separation of destabilized barnase molecules, we co-expressed the optoDroplet construct containing the L14A barnase variant with the Hsp70 protein HSPA1A and/or its cofactor, the Hsp40 protein DNAJB1. The ternary complex is needed for Hsp70 to stimulate ATP hydrolysis and form a high-affinity complex with unfolded proteins. Therefore, the overexpression of Hsp70 alone should result in fewer unfolded proteins being bound by chaperones when compared with Hsp40 alone or Hsp70 overexpressed with Hsp40. Indeed, overexpressing chaperones suppressed droplet formation of L14A barnase (Figure 5D). The overexpression of Hsp70 alone had the smallest effect on suppression of droplet formation, whereas droplets were not observed when Hsp40 was overexpressed or when Hsp70 was jointly overexpressed with Hsp40. Additionally, suppression of droplet formation was more pronounced in the cytoplasm than in the nucleus. This is consistent with overexpressed Hsp70 and Hsp40 accumulating predominantly in the cytoplasm (Figure 5E).

UPODs sequester and enrich cellular proteins through interactions governed by physical chemistry

To understand the physiological consequences of phase separation driven by unfolded molecules, we sought to understand how UPODs engage with the surrounding cellular milieu. Aberrant phase separation may recruit proteostasis machinery and thus modulate the balance of homeostasis (Hipp et al., 2014; Stefani and Dobson, 2003). Further, aberrant phase separation may lead to the sequestration and loss of function of unrelated proteins (Olzscha et al., 2011; Wear et al., 2015). To test for both possibilities, we undertook a compositional profiling of the insoluble fractions of cells, which would be enriched with the UPODs formed by different barnase variants.

We used a proteomics-based strategy to profile the protein compositions of insoluble fractions of cells expressing eight different barnase variants: WT, L14A, Ex4 , 8×A, 3S, 9S, FY, and 4Y (Figures 6A and 6B). HEK293T cells were transfected with each of the eight barnase variants. Cells were lysed gently with non-denaturing buffers, and soluble cytosolic proteins were removed. The compositions of the remaining insoluble material, which retained the variant-specific barnase UPODs, were quantified using mass spectrometry (STAR Methods). We chose barnase variants spanning a range of stabilities, c_{sat} values, and sticker compositions (Figure 6B). In accordance with unfolded molecules forming UPODs, barnase was the most abundant protein in the insoluble fraction for all barnase variants, except WT (Figure S5A). Also, the abundance of barnase was highest for the variants that underwent phase separation (Figure 6C).

Several of the most abundant proteins were found to be chaperones (Figure S5A). To understand how UPODs engage with the surrounding cellular milieu, we examined the abundance of different chaperones in the barnase-specific insoluble fractions. We found that certain chaperones were enriched in a manner that was correlated with the phase separation tendency of the barnase variants (Figure 6C). These chaperones included HSPA1A/B and HSPB1. This suggested that certain chaperones are recruited to UPODs in a way that is non-selective with respect to the sticker compositions of barnase variants. In this case, all variants are likely to be equivalent substrates and the chaperones act in a non-selective manner to maintain the proper balance of folding, binding, and phase equilibria. We also found that other chaperones were enriched in the barnase-specific insoluble fractions in a selective manner that was not correlated with the phase separation tendency of the barnase variants (Figure 6C). These chaperones included CCT7 and HSPA13. Specifically, CCT7 was enriched in the insoluble fractions of the 8×A, FY, and 4Y barnase variants. These variants are all completely unfolded and have exposed aromatic residues. The combination of these features makes 8×A, FY, and 4Y distinct from the other barnase variants and suggests that the accessibility of stickers make them specific substrates for CCT7.

CCT7 is a subunit of the chaperonin-containing T-complex (TRiC) (Spiess et al., 2004). TRiC is composed of eight subunits, namely CCT1–8. All subunits use the same region of the apical domain to interact with substrates (Spiess et al., 2006). For each individual subunit, this region is highly conserved across orthologous subunits (Joachimiak et al., 2014). However, each paralogous subunit has its own sequence composition preferences. These lead to substrate specificity among the CCT subunits. Does the sequence composition of CCT7

(D) Smoothed abundance *Z* score matrix for the top 94 differently enriched proteins in the insoluble fractions (Cox et al., 2022). Here, the *Z* score was calculated using the mean and standard deviation of all replicas and all barnase variants for a given endogenous protein. Proteins were hierarchically clustered using the Euclidean distance and Ward's linkage method. The 24 proteins highlighted in gray are those that were recruited in a manner correlated with the phase separation tendency of the barnase variant.

(E) Significant sequence features in different protein sets. The given set of proteins were significantly enriched in the insoluble fractions of barnase variants to the left of "vs." compared with the barnase variants to the right of "vs." (STAR Methods). Here, "rest" refers to all remaining barnase variants. Features come in three types: patterning, composition, or abundance (STAR Methods). Blue boxes denote either compositional features/abundance that are significantly depleted or patterning features that are well mixed in the given protein set. Red boxes denote either compositional features/abundance that are significantly enriched or patterning features that are blocky in the given protein set. Significance is determined by using the two-sample Kolmogorov-Smirnov test on the *Z* score feature distribution of the given protein set compared with the *Z* score feature distribution of the remaining top 94 proteins. Bar chart shows the number of significantly enriched proteins in each set.

(F) Boxplots of the *Z* scores of the fraction of Arg in proteins significantly enriched in the 4Y insoluble fraction versus rest and fraction of aromatics in proteins significantly enriched in the FY insoluble fraction versus rest. Gray boxplots denote the *Z* scores of the remaining top 94 differently enriched proteins in each case. See also Figure S5 and Table S5.

explain why it targets the 8×A, FY, and 4Y variants? Indeed, the apical domain of CCT7 has the highest fraction of aromatic residues when compared with the other seven subunits (Figure S5C). Additionally, the aromatic residues are localized to the region of the apical domain important for substrate specificity (Figure S5D) (Humphrey et al., 1996; Jumper et al., 2021; Varadi et al., 2022). Thus, it appears that the increased accessibility of aromatic residues in 8×A, FY, and 4Y and the increased aromatic fraction in the substrate recognition region of CCT7 makes these barnase variants specific substrates to CCT7 through interactions involving aromatic residues. This mechanism of engagement is consistent with results showing that mutating a single Trp in the β-isoform of the thromboxane A_2 receptor reduces its interaction with CCT7 (Génier et al., 2016). Overall, our results suggested that certain components of the proteostatic machinery are generically recruited to UPODs to resolve them. However, other chaperones show selectivity based on the barnase variant.

We next asked whether other proteins enriched in the insoluble fractions also showed generic versus selective recruitment. Figure 6D shows the abundance of the top 94 differently enriched endogenous proteins identified by a one-way ANOVA (STAR Methods). Of the 94 proteins, 24 were enriched in the insoluble fractions in a manner that correlated with the underlying phase separation tendency of the barnase variant (Figure 6D, gray solid box). The remaining 70 proteins showed different types of selectivity, including subsets of endogenous proteins that were selectively enriched in insoluble fractions of specific barnase variants (Figure 6D, dashed boxes). We identified proteins that were significantly enriched in a specific barnase insoluble fraction or a set of barnase insoluble fractions. For this, we used a post hoc Fisher's least significant difference (LSD) test following an ANOVA test. For the identified sets of proteins, we did not find statistically significant results in the GO cellular component, GO molecular function, or GO biological process when the entire identified protein set was used as a reference. This result suggested that barnase-specific recruitment was not due to shared cellular functions, processes, or localization among the enriched proteins (Figure S5E) (The UniProt Consortium, 2020; Shemesh et al., 2021; Uhlén et al., 2015). Next, we hypothesized that UPOD-specific recruitment might be due to the physiochemical properties of the proteins, such as complementary interactions with specific stickers that make up each of the barnase variants. To test for this possibility, we extracted ~90 unique sequence features and compared the distribution of these features in each enriched set to the top 94 proteins using the two-sample Kolmogorov-Smirnov test (Figure 6E). We found that recruitment to barnase-specific insoluble fractions depended on the underlying grammar of the specific barnase variant. For example, proteins that were only enriched in the 4Y insoluble fraction showed a higher fraction of Arg residues, and these residues are dispersed uniformly along the linear sequence (Figures 6E and 6F). This is consistent with results showing that the numbers of Tyr and Arg residues jointly contribute to the co-condensation in FET family proteins (Wang et al., 2018). The additional Tyr residues in 4Y might explain why UPODs formed by this variant are enriched in Arg-rich proteins when compared with 8×A and FY. Additionally, for proteins that are only enriched in UPODs formed by the FY variant, we observed an enrichment of proteins with higher fractions of aromatic residues (Figures 6E and 6F). This result is consistent with the fact that Tyr is a stronger sticker than Phe (Bremer et al., 2022).

Taken together, the implication is that UPODs can recruit and sequester cellular proteins through interactions that are governed by physical chemistry alone, without any regard to overlapping or synergistic biological functions. This finding suggests that UPODs might enable gain-of-function interactions that deplete cells of key proteins. It follows that protein-rich deposits that form in the context of disease may have idiopathic effects on toxicity through dysfunction caused by grammar-specific gains of function that are manifest in the form of UPOD-specific compositions.

Sequence grammar that drives the phase separation of unfolded states is similar between barnase and disease-associated IFPs

Do the rules gleaned from studies of barnase transfer to endogenous IFPs from human cells? To answer this question, we performed atomistic simulations of unfolded states for six different unstable IFPs from the human proteome (Figure 1C). We found that all six proteins feature stickers in the unfolded state that are either aliphatic and/or aromatic residues (Figure 7A). These residues account for a large fraction of the total mean contact probability for each protein, and this is larger than what would be expected based purely on their numbers in the sequences. In contrast, while polar residues also account for a large fraction of the total mean contact probability, this is consistent with the number of polar residues in the sequence.

We also examined which residues act as stickers in polyglutamine (polyQ)-expanded Huntingtin exon 1 (Httex1), a protein that is disordered and forms amyloid-like solids in cells (Bäuerlein et al., 2017). In contrast to the IFPs, polar residues dominated the fraction of total mean contact probability in Httex1 with an expanded polyglutamine tract of 49 residues. This fraction was greater than expected, and the result is consistent with studies showing that the phase behavior of Httex1 is driven mainly by amide-amide interactions involving the polyQ domain (Crick et al., 2013; Posey et al., 2018b). These interactions are distinct from the interactions anticipated to be responsible for driving the phase separation of the IFPs studied here.

To test whether IFPs have a similar sticker grammar that is distinct from Httex1, we assessed the colocalization of UPODs formed by the destabilized double mutant of barnase (I25A, I96G, $\Delta G^{\circ}_{U} = -0.8$ kJ/mol) with a destabilizing mutant of SOD1 (A4V) and Httex1 with a glutamine tract of 72 residues (Httex1–72Q). Colocalization would imply that phase separation is governed by similar driving forces. When co-expressed in HEK293T cells, barnase I25A, I96G formed deposits that colocalized with those of SOD1 A4V (Figure 7B). These results imply that phase separation of unfolded barnase and SOD1 are driven by similar interactions. In contrast, the barnase I25A, I96G UPODs did not colocalize with Httex1–72Q deposits (Figure 7B). Previous work has also shown that SOD1 and Httex1 deposits do not colocalize (Farrawell et al., 2015; Polling et al., 2014).

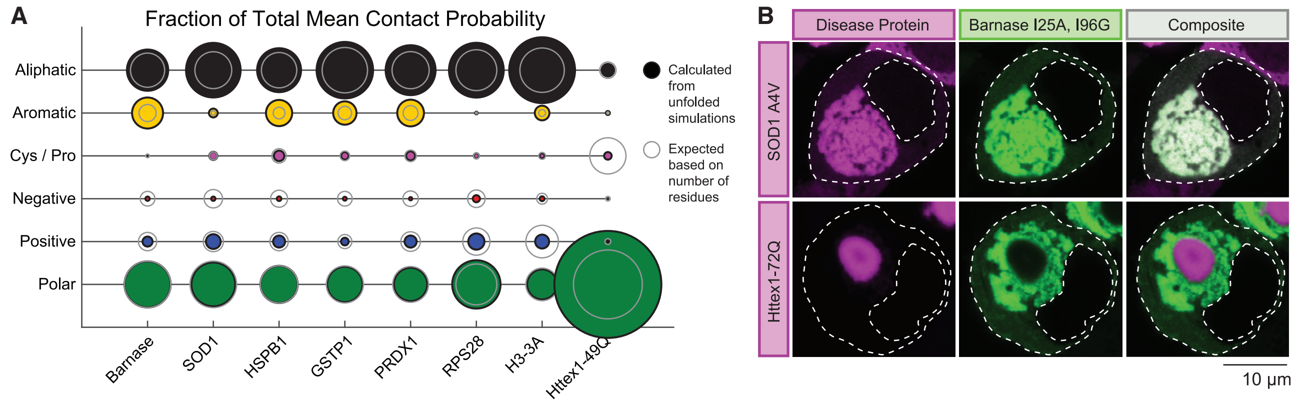

Figure 7. Sequence grammar that drives the phase separation of unfolded states is similar between barnase and disease-associated IFPs
(A) Fraction of total mean contact probability per residue type calculated from atomistic simulations of the unfolded state (STAR Methods).
(B) Fluorescence micrographs show deposits formed by a destabilized variant of barnase (I25A, I96G) flanked with fluorescent proteins (mTFP1 and Venus) (Wood et al., 2018) along with deposits formed by mutant SOD1 (SOD1 A4V) or mutant Httex1 containing a glutamine tract of 72 residues fused to mCherry. The constructs were co-transfected in HEK293T cells. Outlines of cells and nuclei are shown with dashed lines.

This lack of colocalization supports the hypothesis that distinct interactions underlie the phase behavior of SOD1 and barnase variants when compared with Httex1.

DISCUSSION

We have shown that interactions among the unfolded states of IFPs drive intracellular phase separation, leading to the formation of *de novo* UPODs, which is influenced by two features. Phase separation is thermodynamically favored if the protein has a large enough concentration of unfolded proteins *and* has the requisite valence and strength of stickers. The concentration of unfolded proteins is dictated by the free energy of unfolding, whereas the sticker valence and strength are dictated by the composition, accessibility, and sequence contexts in unfolded states.

The specific stickers for IFPs appear to be aliphatic and aromatic residues. Of note, aromatic residues in many intrinsically disordered domains also drive the formation of distinct biomolecular condensates (Frey et al., 2006; Martin et al., 2020). The computational approach we used to identify stickers (Figures 4A and 7A) can be used in conjunction with advances in machine learning (Russ et al., 2020) across the unfolded proteome to make quantitative predictions and identify residues that drive the formation of UPODs.

The driving forces for forming UPODs are modulated by chaperones. Specifically, we found that preferential binding of chaperones to unfolded proteins in the dilute phase leads to a destabilization of UPODs. The modulation of phase separation by the preferential binding of chaperones to the dilute phase represents thermodynamic control through polyphasic linkage (Ruff et al., 2021a, 2021b; Wyman and Gill, 1980) to the regulation of the concentrations of free unfolded proteins. Although the action of Hsp70 involves a combination of preferential binding and ATP hydrolysis, Hsp40 functions purely through preferential binding. Overexpression of Hsp40 has a stronger effect than Hsp70 alone, and their combination has the strongest inhibitory effect on phase separation.

We also found that HSPA1A/B and CCT7 were among the most highly abundant proteins in the insoluble fraction of cells with barnase UPODs (Figure S5A). Unlike CCT7, HSPA1A/B, an Hsp70 protein, is recruited to barnase UPODs in a manner that correlates with phase separation tendency. This suggests that the underlying sequence composition of the substrate has little effect on HSPA1A/B recruitment. However, Hsp70 proteins are often not the first chaperones to bind unfolded substrates. Instead, they are recruited through interactions with other chaperones, including Hsp40s and small heat-shock proteins (sHSPs) such as HSPB1 (Alderson et al., 2016; Veinger et al., 1998). Of note, we found that HSPB1 is also recruited to barnase UPODs in a non-selective way. HSPB1 is an ATP-independent chaperone, and thus its binding to unfolded proteins and modulation of UPOD formation can also be described by polyphasic linkage (Jakob et al., 1993; Ruff et al., 2021a, 2021b). HSPB1 functions by co-assembling with substrates (Gonçalves et al., 2021; Żwirowski et al., 2017). Co-assembly allows for substrates to be held in a proper state needed for Hsp70-dependent disassembly and refolding. Additionally, during this process, Hsp70 and its co-chaperones remove sHsps from the assembly. This process might explain why HSPA1A/B is more abundant in UPODs than HSPB1. Overall, our results suggest that UPODs may be generally targeted by sHSPs in collaboration with Hsp70 to modulate the formation of UPODs and refold IFPs, consistent with the effects of HSPB1 and Hsp70 on SOD1 phase separation and ALS progression (Patel et al., 2005; Sharp et al., 2008; Yerbury et al., 2013).

The shared chaperone regulation pathway between the model protein barnase and a human disease-related IFP suggests that the features that influence recruitment into UPODs formed by barnase variants are likely to be transferrable to other IFPs. Of interest is the observation that proteins can be recruited to UPODs based on a shared grammar for interactions of cellular proteins with the unfolded states of the phase separating IFP. Indeed, it is known that Httex1 with expanded polyglutamine tracts recruits proteins with long IDRs into its deposits (Wear et al.,

2015). Deletion of the long IDRs in two of the recruited proteins decreases colocalization with Httex1. These results suggest that IDR-IDR interactions between Httex1 and other cellular proteins may lead to sequestration and subsequent loss of function of recruited proteins.

The recruitment of proteins based on shared interaction grammars implies that the relevant residues must be accessible for heterotypic interactions. Residues may be accessible if they are part of an IDR. However, 66 of the top 94 differently enriched proteins (70%) do not contain an IDR of a length greater than 50 amino acids. Instead, residues may be accessible if they are sequestered in UPODs before they have the chance to fold. If newly synthesized proteins are also preferentially recruited to UPODs, then proteins that require long time scales to fold or the help of many chaperones might be susceptible to recruitment into aberrant UPODs. Both Hsp70 and TRiC can work together for co-translational folding of substrates (Stein et al., 2019). Thus, the recruitment of these chaperones to UPODs may further increase the population of unfolded or improperly folded newly synthesized proteins.

Kaganovich et al. identified two protein quality control compartments named the insoluble protein deposit (IPOD) and juxtanuclear quality control compartment (JUNQ) (Kaganovich et al., 2008). Polyglutamine-containing proteins formed IPODs (Kaganovich et al., 2008), whereas other misfolded proteins, such as SOD1-destabilizing variants, formed JUNQs (Polling et al., 2014). The colocalization of the I25A, I96G barnase variant with SOD1 and the enrichment of HSPA1A/B in barnase UPODs suggests that UPODs may be equivalent to JUNQ compartments (Weisberg et al., 2012). If the two compartments are equivalent, then our compositional profiling data would suggest the compositions of JUNQs are unique to the sticker grammars of unfolded/misfolded IFP(s) that drive its formation. Differences in composition could lead to differences in cell-specific stresses. Therefore, determining the relationship between UPODs and JUNQs is important for understanding how cells manage unfolded/misfolded IFPs.

Limitations of the study

We explored UPOD formation by modulating the expression levels of the unfolded protein molecules and assumed that the homotypic interactions among these molecules are the dominant interactions that drive UPOD formation. However, ligands and other heterotypic interactions can also influence the threshold concentrations for phase separation. Finally, the impacts of the rates of folding-unfolding, the contributions of folding intermediates—and hence partially folded/unfolded states—and the roles of cellular states on UPOD formation were not part of this study.

STAR★METHODS

Detailed methods are provided in the online version of this paper and include the following:

- KEY RESOURCES TABLE
- RESOURCE AVAILABILITY
  - Lead contact
  - Materials availability
  - Data and code availability
- EXPERIMENTAL MODEL AND SUBJECT DETAILS
  - Cell lines
- METHOD DETAILS
  - Cell imaging
  - Constructs
  - Image analysis
  - Extraction of c_{sat} values for optoDroplet formation of barnase
  - Fitting of c_{sat} versus ΔG°_{U}
  - c^{*} confidence interval
  - Computational mutagenesis study predicting ΔG°_{U}
  - Atomistic simulations
  - Identifying stickers from atomistic simulations of unfolded states
  - Flow cytometry
  - LC-MS/MS sample preparation and analysis
  - Assignment of functional categories
  - Sequence feature analysis
  - Disorder analysis
- QUANTIFICATION AND STATISTICAL ANALYSIS

SUPPLEMENTAL INFORMATION

Supplemental information can be found online at https://doi.org/10.1016/j.molcel.2022.06.024.

ACKNOWLEDGMENTS

We are grateful to our colleagues and collaborators Chloe Gerak, Mina Farag, Beatriz Ferreira-Gomes, Marta Frigole-Vivas, Paul Gooley, Matthew King, Tanja Mittag, Ammon Posey, and Min Kyung Shinn for helpful discussions. This work was supported by grants APP1161803 and APP1154352 from the National Health and Medical Research Council of Australia, grant DP170103093 from the Australian Research Council, the US National Institutes of Health (5R01NS056114), the US Air Force Office of Scientific Research (FA9550-20-1-0241), and the Wellcome Trust (204963).

AUTHOR CONTRIBUTIONS

Y.H.C., D.C., and A.R.O. performed measurements. K.M.R., R.V.P., and D.M.H. designed the analysis. K.M.R. performed the simulations and computational analysis. Y.M. and D.B.A. generated the estimates of stabilities for barnase variants. S.E.R. and D.C. helped with variant design. K.M.R., Y.H.C., D.C., and A.R.O. made the figures. K.M.R., D.M.H., and R.V.P. wrote and edited the first full drafts of the manuscript. All authors revised the manuscript.

DECLARATION OF INTERESTS

R.V.P. is a member of the Scientific Advisory Board of Dewpoint Therapeutics Inc.

INCLUSION AND DIVERSITY

One or more of the authors of this paper self-identifies as an underrepresented ethnic minority in science. One or more of the authors of this paper self-identifies as a member of the LGBTQ+ community.

Received: August 19, 2021
Revised: May 5, 2022
Accepted: June 15, 2022
Published: July 18, 2022

REFERENCES

Alderson, T.R., Kim, J.H., and Markley, J.L. (2016). Dynamical structures of Hsp70 and Hsp70-Hsp40 complexes. Structure *24*, 1014–1030.

Arrasate, M., Mitra, S., Schweitzer, E.S., Segal, M.R., and Finkbeiner, S. (2004). Inclusion body formation reduces levels of mutant huntingtin and the risk of neuronal death. Nature *431*, 805–810.

Balch, W.E., Morimoto, R.I., Dillin, A., and Kelly, J.W. (2008). Adapting proteostasis for disease intervention. Science *319*, 916–919.

Balchin, D., Hayer-Hartl, M., and Hartl, F.U. (2020). Recent advances in understanding catalysis of protein folding by molecular chaperones. FEBS Lett. *594*, 2770–2781.

Bäuerlein, F.J.B., Saha, I., Mishra, A., Kalemanov, M., Martínez-Sánchez, A., Klein, R., Dudanova, I., Hipp, M.S., Hartl, F.U., Baumeister, W., and Fernández-Busnadiego, R. (2017). In situ architecture and cellular interactions of PolyQ inclusions. Cell *171*, 179–187.e10.

Bobori, C., Theocharopoulou, G., and Vlamos, P. (2017). Molecular chaperones in neurodegenerative diseases: a short review. Adv. Exp. Med. Biol. (Springer International Publishing) *987*, 219–231.

Brady, J.P., Farber, P.J., Sekhar, A., Lin, Y.-H., Huang, R., Bah, A., Nott, T.J., Chan, H.S., Baldwin, A.J., Forman-Kay, J.D., and Kay, L.E. (2017). Structural and hydrodynamic properties of an intrinsically disordered region of a germ cell-specific protein on phase separation. Proc. Natl. Acad. Sci. USA *114*, E8194–E8203.

Bremer, A., Farag, M., Borcherds, W.M., Peran, I., Martin, E.W., Pappu, R.V., and Mittag, T. (2022). Deciphering how naturally occurring sequence features impact the phase behaviours of disordered prion-like domains. Nat. Chem. *14*, 196–207.

Choi, J.-M., Holehouse, A.S., and Pappu, R.V. (2020). Physical principles underlying the complex biology of intracellular phase transitions. Annu. Rev. Biophys. *49*, 107–133.

Ciryam, P., Lambert-Smith, I.A., Bean, D.M., Freer, R., Cid, F., Tartaglia, G.G., Saunders, D.N., Wilson, M.R., Oliver, S.G., Morimoto, R.I., et al. (2017). Spinal motor neuron protein supersaturation patterns are associated with inclusion body formation in ALS. Proc. Natl. Acad. Sci. USA *114*, E3935–E3943.

Ciryam, P., Tartaglia, G.G., Morimoto, R.I., Dobson, C.M., and Vendruscolo, M. (2013). Widespread aggregation and neurodegenerative diseases are associated with supersaturated proteins. Cell Rep. *5*, 781–790.

Clark, P.L. (2004). Protein folding in the cell: reshaping the folding funnel. Trends Biochem. Sci. *29*, 527–534.

Cohan, M.C., Shinn, M.K., Lalmansingh, J.M., and Pappu, R.V. (2022). Uncovering non-random binary patterns Within sequences of intrinsically disordered proteins. J. Mol. Biol. *434*, 167373.

Cook, R.D. (1977). Detection of influential observation in linear regression. Technometrics *19*, 15–18.

Cox, D., Ang, C.-S., Nillegoda, N.B., Reid, G.E., and Hatters, D.M. (2022). Hidden information on protein function in censuses of proteome foldedness. Nat. Commun. *13*, 1992.

Cox, J., and Mann, M. (2008). MaxQuant enables high peptide identification rates, individualized p.p.b.-range mass accuracies and proteome-wide protein quantification. Nat. Biotechnol. *26*, 1367–1372.

Crick, S.L., Jayaraman, M., Frieden, C., Wetzel, R., and Pappu, R.V. (2006). Fluorescence correlation spectroscopy shows that monomeric polyglutamine molecules form collapsed structures in aqueous solutions. Proc. Natl. Acad. Sci. USA *103*, 16764–16769.

Crick, S.L., Ruff, K.M., Garai, K., Frieden, C., and Pappu, R.V. (2013). Unmasking the roles of N- and C-terminal flanking sequences from exon 1 of huntingtin as modulators of polyglutamine aggregation. Proc. Natl. Acad. Sci. USA *110*, 20075–20080.

Dalby, P.A., Oliveberg, M., and Fersht, A.R. (1998). Movement of the intermediate and rate determining transition state of barnase on the energy landscape with changing temperature. Biochemistry *37*, 4674–4679.

Danielsson, J., Mu, X., Lang, L., Wang, H., Binolfi, A., Theillet, F.-X., Bekei, B., Logan, D.T., Selenko, P., Wennerström, H., and Oliveberg, M. (2015). Thermodynamics of protein destabilization in live cells. Proc. Natl. Acad. Sci. USA *112*, 12402–12407.

Farrawell, N.E., Lambert-Smith, I.A., Warraich, S.T., Blair, I.P., Saunders, D.N., Hatters, D.M., and Yerbury, J.J. (2015). Distinct partitioning of ALS associated TDP-43, FUS and SOD1 mutants into cellular inclusions. Sci. Rep. *5*, 13416.

Frey, S., Richter, R.P., and Görlich, D. (2006). FG-rich repeats of nuclear pore proteins form a three-dimensional meshwork with hydrogel-like properties. Science *314*, 815–817.

Garai, K., Sahoo, B., Sengupta, P., and Maiti, S. (2008). Quasihomogeneous nucleation of amyloid beta yields numerical bounds for the critical radius, the surface tension, and the free energy barrier for nucleus formation. J. Chem. Phys. *128*, 045102.

Génier, S., Degrandmaison, J., Moreau, P., Labrecque, P., Hébert, T.E., and Parent, J.-L. (2016). Regulation of GPCR expression through an interaction with CCT7, a subunit of the CCT/TRiC complex. Mol. Biol. Cell *27*, 3800–3812.

Gnutt, D., Timr, S., Ahlers, J., König, B., Manderfeld, E., Heyden, M., Sterpone, F., and Ebbinghaus, S. (2019). Stability effect of quinary interactions reversed by single point mutations. J. Am. Chem. Soc. *141*, 4660–4669.

Gomez, M., and Germain, D. (2019). Cross talk between SOD1 and the mitochondrial UPR in cancer and neurodegeneration. Mol. Cell. Neurosci. *98*, 12–18.

Gonçalves, C.C., Sharon, I., Schmeing, T.M., Ramos, C.H.I., and Young, J.C. (2021). The chaperone HSPB1 prepares protein aggregates for resolubilization by HSP70. Sci. Rep. *11*, 17139.

Hageman, J., and Kampinga, H.H. (2009). Computational analysis of the human HSPH/HSPA/DNAJ family and cloning of a human HSPH/HSPA/DNAJ expression library. Cell Stress Chaperones *14*, 1–21.

Hartl, F.U. (2016). Cellular homeostasis and aging. Annu. Rev. Biochem. *85*, 1–4.

Hipp, M.S., Park, S.-H., and Hartl, F.U. (2014). Proteostasis impairment in protein-misfolding and -aggregation diseases. Trends Cell Biol. *24*, 506–514.

Holehouse, A.S., Das, R.K., Ahad, J.N., Richardson, M.O.G., and Pappu, R.V. (2017). CIDER: resources to analyze sequence-ensemble relationships of intrinsically disordered proteins. Biophys. J. *112*, 16–21.

Holehouse, A.S., Garai, K., Lyle, N., Vitalis, A., and Pappu, R.V. (2015). Quantitative assessments of the distinct contributions of polypeptide backbone amides versus side chain groups to chain expansion via chemical denaturation. J. Am. Chem. Soc. *137*, 2984–2995.

Hsu, D.S., Zhao, X., Zhao, S., Kazantsev, A., Wang, R.-P., Todo, T., Wei, Y.-F., and Sancar, A. (1996). Putative human blue-light photoreceptors hCRY1 and hCRY2 are flavoproteins. Biochemistry *35*, 13871–13877.

Humphrey, W., Dalke, A., and Schulten, K. (1996). VMD: visual molecular dynamics. J. Mol. Graph. *14*, 33–38.

Jakob, U., Gaestel, M., Engel, K., and Buchner, J. (1993). Small heat shock proteins are molecular chaperones. J. Biol. Chem. *268*, 1517–1520.

Jiang, Y., Rossi, P., and Kalodimos, C.G. (2019). Structural basis for client recognition and activity of Hsp40 chaperones. Science *365*, 1313–1319.

Joachimiak, L.A., Walzthoeni, T., Liu, C.W., Aebersold, R., and Frydman, J. (2014). The structural basis of substrate recognition by the eukaryotic chaperonin TRiC/CCT. Cell *159*, 1042–1055.

Jumper, J., Evans, R., Pritzel, A., Green, T., Figurnov, M., Ronneberger, O., Tunyasuvunakool, K., Bates, R., Žídek, A., Potapenko, A., et al. (2021). Highly accurate protein structure prediction with AlphaFold. Nature *596*, 583–589.

Kaganovich, D., Kopito, R., and Frydman, J. (2008). Misfolded proteins partition between two distinct quality control compartments. Nature *454*, 1088–1095.

Kanehisa, M., and Goto, S. (2000). KEGG: Kyoto encyclopedia of genes and genomes. Nucleic Acids Res. *28*, 27–30.

CellPress
OPEN ACCESS

Kyte, J., and Doolittle, R.F. (1982). A simple method for displaying the hydropathic character of a protein. J. Mol. Biol. *157*, 105–132.

Lang, L., Zetterström, P., Brännström, T., Marklund, S.L., Danielsson, J., and Oliveberg, M. (2015). SOD1 aggregation in ALS mice shows simplistic test tube behavior. Proc. Natl. Acad. Sci. U. S. A. *112*, 9878–9883.

Leuenberger, P., Ganscha, S., Kahraman, A., Cappelletti, V., Boersema, P.J., von Mering, C.v., Claassen, M., and Picotti, P. (2017). Cell-wide analysis of protein thermal unfolding reveals determinants of thermostability. Science *355*. eaai7825.

Li, C., Wen, A., Shen, B., Lu, J., Huang, Y., and Chang, Y. (2011). FastCloning: a highly simplified, purification-free, sequence- and ligation-independent PCR cloning method. BMC Biotechnol. *11*, 92.

Lin, C., Yang, H., Guo, H., Mockler, T., Chen, J., and Cashmore, A.R. (1998). Enhancement of blue-light sensitivity of Arabidopsis seedlings by a blue light receptor cryptochrome 2. Proc. Natl. Acad. Sci. U. S. A. *95*, 2686–2690.

Lin, Y., Currie, S.L., and Rosen, M.K. (2017). Intrinsically disordered sequences enable modulation of protein phase separation through distributed tyrosine motifs. J. Biol. Chem. *292*, 19110–19120.

Lohia, R., Salari, R., and Brannigan, G. (2019). Sequence specificity despite intrinsic disorder: how a disease-associated Val/Met polymorphism rearranges tertiary interactions in a long disordered protein. PLOS Comput. Biol. *15*, e1007390.

Maier, E.M., Gersting, S.W., Kemter, K.F., Jank, J.M., Reindl, M., Messing, D.D., Truger, M.S., Sommerhoff, C.P., and Muntau, A.C. (2009). Protein misfolding is the molecular mechanism underlying MCADD identified in newborn screening. Hum. Mol. Genet. *18*, 1612–1623.

Martin, E.W., Holehouse, A.S., Peran, I., Farag, M., Incicco, J.J., Bremer, A., Grace, C.R., Soranno, A., Pappu, R.V., and Mittag, T. (2020). Valence and patterning of aromatic residues determine the phase behavior of prion-like domains. Science *367*, 694–699.

Mathieu, C., Pappu, R.V., and Taylor, J.P. (2020). Beyond aggregation: pathological phase transitions in neurodegenerative disease. Science *370*, 56–60.

Matthews, J.M., and Fersht, A.R. (1995). Exploring the energy surface of protein folding by structure-reactivity relationships and engineered proteins: observation of Hammond behavior for the gross structure of the transition state and anti-Hammond behavior for structural elements for unfolding/folding of barnase. Biochemistry *34*, 6805–6814.

Meiering, E.M. (2008). The threat of instability: neurodegeneration predicted by protein destabilization and aggregation propensity. PLOS Biol. *6*, e193.

Mi, H., Ebert, D., Muruganujan, A., Mills, C., Albou, L.P., Mushayamaha, T., and Thomas, P.D. (2021). PANTHER version 16: a revised family classification, tree-based classification tool, enhancer regions and extensive API. Nucleic Acids Res. *49*, D394–D403.

Nikam, R., Kulandaisamy, A., Harini, K., Sharma, D., and Gromiha, M.M. (2021). ProThermDB: thermodynamic database for proteins and mutants revisited after 15 years. Nucleic Acids Res. *49*, D420–D424.

Nordlund, A., Leinartaitė, L., Saraboji, K., Aisenbrey, C., Gröbner, G., Zetterström, P., Danielsson, J., Logan, D.T., and Oliveberg, M. (2009). Functional features cause misfolding of the ALS-provoking enzyme SOD1. Proc. Natl. Acad. Sci. USA *106*, 9667–9672.

Nott, T.J., Petsalaki, E., Farber, P., Jervis, D., Fussner, E., Plochowietz, A., Craggs, T.D., Bazett-Jones, D.P., Pawson, T., Forman-Kay, J.D., and Baldwin, A.J. (2015). Phase transition of a disordered nuage protein generates environmentally responsive membraneless organelles. Mol. Cell *57*, 936–947.

Olzscha, H., Schermann, S.M., Woerner, A.C., Pinkert, S., Hecht, M.H., Tartaglia, G.G., Vendruscolo, M., Hayer-Hartl, M., Hartl, F.U., and Vabulas, R.M. (2011). Amyloid-like aggregates sequester numerous metastable proteins with essential cellular functions. Cell *144*, 67–78.

Ormsby, A.R., Ramdzan, Y.M., Mok, Y.-F., Jovanoski, K.D., and Hatters, D.M. (2013). A platform to view huntingtin exon 1 aggregation flux in the cell reveals divergent influences from chaperones hsp40 and hsp70. J. Biol. Chem. *288*, 37192–37203.

Pang, Z., Chong, J., Zhou, G., Morais, D., Chang, L., Barrette, M., Gauthier, C., Jacques, P.E., Li, S., and Xia, J. (2021). MetaboAnalyst 5.0: narrowing the gap between raw spectra and functional insights. Nucleic Acids Res. *49*, W388–W396.

Pappu, R.V., Wang, X., Vitalis, A., and Crick, S.L. (2008). A polymer physics perspective on driving forces and mechanisms for protein aggregation. Arch. Biochem. Biophys. *469*, 132–141.

Patel, Y.J.K., Payne Smith, M.D., de Belleroche, J., and Latchman, D.S. (2005). Hsp27 and Hsp70 administered in combination have a potent protective effect against FALS-associated SOD1-mutant-induced cell death in mammalian neuronal cells. Brain Res. Mol. Brain Res. *134*, 256–274.

Peran, I., Holehouse, A.S., Carrico, I.S., Pappu, R.V., Bilsel, O., and Raleigh, D.P. (2019). Unfolded states under folding conditions accommodate sequence-specific conformational preferences with random coil-like dimensions. Proc. Natl. Acad. Sci. USA *116*, 12301–12310.

Piovesan, D., Necci, M., Escobedo, N., Monzon, A.M., Hatos, A., Mičetić, I., Quaglia, F., Paladin, L., Ramasamy, P., Dosztányi, Z., et al. (2021). MobiDB: intrinsically disordered proteins in 2021. Nucleic Acids Res. *49*, D361–D367.

Polling, S., Mok, Y.-F., Ramdzan, Y.M., Turner, B.J., Yerbury, J.J., Hill, A.F., and Hatters, D.M. (2014). Misfolded polyglutamine, polyalanine, and superoxide dismutase 1 aggregate via distinct pathways in the cell. J. Biol. Chem. *289*, 6669–6680.

Posey, A.E., Holehouse, A.S., and Pappu, R.V. (2018a). Chapter One. Phase separation of intrinsically disordered proteins. In Methods in Enzymology, E. Rhoades, ed. (Academic Press), pp. 1–30.

Posey, A.E., Ruff, K.M., Harmon, T.S., Crick, S.L., Li, A., Diamond, M.I., and Pappu, R.V. (2018b). Profilin reduces aggregation and phase separation of huntingtin N-terminal fragments by preferentially binding to soluble monomers and oligomers. J. Biol. Chem. *293*, 3734–3746.

Powers, E.T., Morimoto, R.I., Dillin, A., Kelly, J.W., and Balch, W.E. (2009). Biological and chemical approaches to diseases of proteostasis deficiency. Annu. Rev. Biochem. *78*, 959–991.

Reinle, K., Mogk, A., and Bukau, B. (2022). The diverse functions of small heat shock proteins in the proteostasis network. J. Mol. Biol. *434*, 167157.

Ruff, K.M., Dar, F., and Pappu, R.V. (2021a). Ligand effects on phase separation of multivalent macromolecules. Proc. Natl. Acad. Sci. USA *118*, e2017184118.

Ruff, K.M., Dar, F., and Pappu, R.V. (2021b). Polyphasic linkage and the impact of ligand binding on the regulation of biomolecular condensates. Biophys. Rev. *2*, 021302.

Russ, W.P., Figliuzzi, M., Stocker, C., Barrat-Charlaix, P., Socolich, M., Kast, P., Hilvert, D., Monasson, R., Cocco, S., Weigt, M., and Ranganathan, R. (2020). An evolution-based model for designing chorismate mutase enzymes. Science *369*, 440–445.

Ryno, L.M., Wiseman, R.L., and Kelly, J.W. (2013). Targeting unfolded protein response signaling pathways to ameliorate protein misfolding diseases. Curr. Opin. Chem. Biol. *17*, 346–352.

Schindelin, J., Arganda-Carreras, I., Frise, E., Kaynig, V., Longair, M., Pietzsch, T., Preibisch, S., Rueden, C., Saalfeld, S., Schmid, B., et al. (2012). Fiji: an open-source platform for biological-image analysis. Nat. Methods *9*, 676–682.

Sharp, P.S., Akbar, M.T., Bouri, S., Senda, A., Joshi, K., Chen, H.-J., Latchman, D.S., Wells, D.J., and de Belleroche, J. (2008). Protective effects of heat shock protein 27 in a model of ALS occur in the early stages of disease progression. Neurobiol. Dis. *30*, 42–55.

Shemesh, N., Jubran, J., Dror, S., Simonovsky, E., Basha, O., Argov, C., Hekselman, I., Abu-Qarn, M., Vinogradov, E., Mauer, O., et al. (2021). The landscape of molecular chaperones across human tissues reveals a layered architecture of core and variable chaperones. Nat. Commun. *12*, 2180.

Shin, Y., Berry, J., Pannucci, N., Haataja, M.P., Toettcher, J.E., and Brangwynne, C.P. (2017). Spatiotemporal control of intracellular phase transitions using light-activated optoDroplets. Cell *168*, 159–171.e14.

Solomon, J.P., Page, L.J., Balch, W.E., and Kelly, J.W. (2012). Gelsolin amyloidosis: genetics, biochemistry, pathology and possible strategies for therapeutic intervention. Crit. Rev. Biochem. Mol. Biol. *47*, 282–296.

Song, J. (2018). Environment-transformable sequence–structure relationship: a general mechanism for proteotoxicity. Biophys. Rev. *10*, 503–516.

Sontag, E.M., Samant, R.S., and Frydman, J. (2017). Mechanisms and functions of spatial protein quality control. Annu. Rev. Biochem. *86*, 97–122.

Sormanni, P., Aprile, F.A., and Vendruscolo, M. (2015). The CamSol method of rational design of protein mutants with enhanced solubility. J. Mol. Biol. *427*, 478–490.

Spiess, C., Meyer, A.S., Reissmann, S., and Frydman, J. (2004). Mechanism of the eukaryotic chaperonin: protein folding in the chamber of secrets. Trends Cell Biol. *14*, 598–604.

Spiess, C., Miller, E.J., McClellan, A.J., and Frydman, J. (2006). Identification of the TRiC/CCT substrate binding sites uncovers the function of subunit diversity in eukaryotic chaperonins. Mol. Cell *24*, 25–37.

Stefani, M., and Dobson, C.M. (2003). Protein aggregation and aggregate toxicity: new insights into protein folding, misfolding diseases and biological evolution. J. Mol. Med. (Berl) *81*, 678–699.

Stein, K.C., Kriel, A., and Frydman, J. (2019). Nascent polypeptide domain topology and elongation rate direct the cotranslational hierarchy of Hsp70 and TRiC/CCT. Mol. Cell *75*, 1117–1130.e5.

Stringer, C., Wang, T., Michaelos, M., and Pachitariu, M. (2021). Cellpose: a generalist algorithm for cellular segmentation. Nat. Methods *18*, 100–106.

Turner, B.J., Atkin, J.D., Farg, M.A., Zang, D.W., Rembach, A., Lopes, E.C., Patch, J.D., Hill, A.F., and Cheema, S.S. (2005). Impaired extracellular secretion of mutant superoxide dismutase 1 associates with neurotoxicity in familial amyotrophic lateral sclerosis. J. Neurosci. *25*, 108–117.

Tyson, A.L., Rousseau, C.V., Niedworok, C.J., Keshavarzi, S., Tsitoura, C., Cossell, L., Strom, M., and Margrie, T.W. (2021). A deep learning algorithm for 3D cell detection in whole mouse brain image datasets. PLOS Comput. Biol. *17*, e1009074.

Uhlén, M., Fagerberg, L., Hallström, B.M., Lindskog, C., Oksvold, P., Mardinoglu, A., Sivertsson, Å., Kampf, C., Sjöstedt, E., Asplund, A., et al. (2015). Tissue-based map of the human proteome. Science *347*, 1260419.

The UniProt Consortium (2020). UniProt: the universal protein KnowledgeBase in 2021. Nucleic Acids Res. *49*, D480–D489.

Varadi, M., Anyango, S., Deshpande, M., Nair, S., Natassia, C., Yordanova, G., Yuan, D., Stroe, O., Wood, G., Laydon, A., et al. (2022). AlphaFold Protein Structure Database: massively expanding the structural coverage of protein-sequence space with high-accuracy models. Nucleic Acids Res. *50*, D439–D444.

Veinger, L., Diamant, S., Buchner, J., and Goloubinoff, P. (1998). The small heat-shock protein IbpB from *Escherichia coli* stabilizes stress-denatured proteins for subsequent refolding by a multichaperone network. J. Biol. Chem. *273*, 11032–11037.

Vendruscolo, M., and Dobson, C.M. (2005). Towards complete descriptions of the free-energy landscapes of proteins. Phil. Trans. R. Soc. A. *363*, 433–452.

Vitalis, A., and Pappu, R.V. (2009). ABSINTH: A new continuum solvation model for simulations of polypeptides in aqueous solutions. J. Comput. Chem. *30*, 673–699.

Wang, J., Choi, J.-M., Holehouse, A.S., Lee, H.O., Zhang, X., Jahnel, M., Maharana, S., Lemaitre, R., Pozniakovsky, A., Drechsel, D., et al. (2018). A molecular grammar governing the driving forces for phase separation of prion-like RNA binding proteins. Cell *174*, 688–699.e16.

Warner, J.B., Ruff, K.M., Tan, P.S., Lemke, E.A., Pappu, R.V., and Lashuel, H.A. (2017). Monomeric huntingtin exon 1 has similar overall structural features for wild-type and pathological polyglutamine lengths. J. Am. Chem. Soc. *139*, 14456–14469.

Wear, M.P., Kryndushkin, D., O'Meally, R., Sonnenberg, J.L., Cole, R.N., and Shewmaker, F.P. (2015). Proteins with intrinsically disordered domains are preferentially recruited to polyglutamine aggregates. PLoS One *10*, e0136362.

Weisberg, S.J., Lyakhovetsky, R., Werdiger, A.-c., Gitler, A.D., Soen, Y., and Kaganovich, D. (2012). Compartmentalization of superoxide dismutase 1 (SOD1G93A) aggregates determines their toxicity. Proc. Natl. Acad. Sci. USA *109*, 15811–15816.

Wood, R.J., Ormsby, A.R., Radwan, M., Cox, D., Sharma, A., Vöpel, T., Ebbinghaus, S., Oliveberg, M., Reid, G.E., Dickson, A., and Hatters, D.M. (2018). A biosensor-based framework to measure latent proteostasis capacity. Nat. Commun. *9*, 287.

Wyman, J., and Gill, S.J. (1980). Ligand-linked phase changes in a biological system: applications to sickle cell hemoglobin. Proc. Natl. Acad. Sci. USA *77*, 5239–5242.

Yerbury, J.J., Gower, D., Vanags, L., Roberts, K., Lee, J.A., and Ecroyd, H. (2013). The small heat shock proteins αB-crystallin and Hsp27 suppress SOD1 aggregation in vitro. Cell Stress Chaperones *18*, 251–257.

Zarin, T., Strome, B., Nguyen Ba, A.N., Alberti, S., Forman-Kay, J.D., and Moses, A.M. (2019). Proteome-wide signatures of function in highly diverged intrinsically disordered regions. eLife *8*, e46883.

Zeng, X., Holehouse, A.S., Chilkoti, A., Mittag, T., and Pappu, R.V. (2020). Connecting coil-to-globule transitions to full phase diagrams for intrinsically disordered proteins. Biophys. J. *119*, 402–418.

Żwirowski, S., Kłosowska, A., Obuchowski, I., Nillegoda, N.B., Piróg, A., Ziętkiewicz, S., Bukau, B., Mogk, A., and Liberek, K. (2017). Hsp70 displaces small heat shock proteins from aggregates to initiate protein refolding. EMBO J. *36*, 783–796.

CellPress
OPEN ACCESS

STAR★METHODS

KEY RESOURCES TABLE

REAGENT or RESOURCE	SOURCE	IDENTIFIER
**Antibodies**		
Anti-V5 antibody	Abcam	Cat#Ab27671; RRID:AB_471093
Anti-HSPA1A antibody	Abcam	Cat#Ab5439; RRID:AB_304888
Goat anti-mouse Cyanine5	Life technologies	Cat#A10524; RRID:AB_2534033
**Chemicals, peptides, and recombinant proteins**		
VER-155008	Sigma-Aldrich	Cat#SML0271
Opti-MEM	Thermo Fisher Scientific	Cat#31985062
Dulbecco’s Modified Eagle Medium	Thermo Fisher Scientific	Cat#21013024
Fetal bovine serum	Thermo Fisher Scientific	Cat#10099141
L-glutamine	Thermo Fisher Scientific	Cat#25030081
Lipofectamine 3000	Thermo Fisher Scientific	Cat#L3000015
Hoechst 33342	Thermo Fisher Scientific	Cat#H3570
Hank’s Balanced Salt Solution	Thermo Fisher Scientific	Cat#14025092
Digitonin	Sigma-Aldrich	Cat#D141
Complete EDTA-free protease inhibitor cocktail	Sigma-Aldrich	Cat#11836170001
Pierce Trypsin protease, MS grade	Thermo Fisher Scientific	Cat#90057
TMT10plex Isobaric label reagent set plus TMT11-131C label reagent	Thermo Fisher Scientific	Cat#A34808
**Critical commercial assays**		
BCA protein assay	Thermo Fisher Scientific	Cat#23227
**Deposited data**		
Raw proteomics data	ProteomeXchange Consortium	PRIDE: PXD033716
Raw and analyzed data	This paper; Zenodo	Zenodo data: https://doi.org/10.5281/zenodo.6603909
Raw image data used to assemble figures	This paper; Mendeley	Mendeley data: https://doi.org/10.17632/3rrp4zy7p3.1
**Experimental models: Cell lines**		
Mouse: Neuro2a cells	American Type Culture Collection	Cat#CCL-131
Human: HEK293T cells	Laboratory of Greg Mosely, Monash University, Australia	N/A
**Recombinant DNA**		
DDX4 IDR–mCherry–Cry2	Shin et al., 2017	N/A
Barnase WT– mCherry–Cry2	This paper	N/A
Barnase I55G– mCherry–Cry2	This paper	N/A
Barnase V45T– mCherry–Cry2	This paper	N/A
Barnase I25A– mCherry–Cry2	This paper	N/A
Barnase I88A– mCherry–Cry2	This paper	N/A
Barnase L14A– mCherry–Cry2	This paper	N/A
Barnase I51A– mCherry–Cry2	This paper	N/A
Barnase L89G– mCherry–Cry2	This paper	N/A
Barnase I96G– mCherry–Cry2	This paper	N/A
Barnase I55G, L89G– mCherry–Cry2	This paper	N/A
Barnase I88G– mCherry–Cry2	This paper	N/A

(*Continued on next page*)

Continued		
REAGENT or RESOURCE	SOURCE	IDENTIFIER
Barnase V45T, I88G– mCherry–Cry2	This paper	N/A
Barnase I25A, I96G– mCherry–Cry2	This paper	N/A
Barnase L14A, I51A, I88A– mCherry–Cry2	This paper	N/A
Barnase L14G, I51G, I88G– mCherry–Cry2	This paper	N/A
Barnase L14S, I51S, I88S– mCherry–Cry2	This paper	N/A
Barnase L14D, I51D, I88D– mCherry–Cry2	This paper	N/A
Barnase 8×A–mCherry–Cry2	This paper	N/A
Barnase 8×G–mCherry–Cry2	This paper	N/A
Barnase 8×S–mCherry–Cry2	This paper	N/A
Barnase 8×D–mCherry–Cry2	This paper	N/A
Barnase 9S–mCherry–Cry2	This paper	N/A
Barnase 7S–mCherry–Cry2	This paper	N/A
Barnase 5S–mCherry–Cry2	This paper	N/A
Barnase 3S–mCherry–Cry2	This paper	N/A
Barnase F7Y–mCherry–Cry2	This paper	N/A
Barnase F106Y–mCherry–Cry2	This paper	N/A
Barnase F7Y, F106Y–mCherry–Cry2	This paper	N/A
Barnase F7Y, F82Y, F106Y–mCherry–Cry2	This paper	N/A
Barnase FY–mCherry–Cry2	This paper	N/A
Barnase 4Y–mCherry–Cry2	This paper	N/A
mTFP1–Barnase WT–Venus	Wood et al., 2018	N/A
mTFP1–Barnase L14A–Venus	Wood et al., 2018	N/A
mTFP1–Barnase I25A, I96G–Venus	Wood et al., 2018	N/A
mTFP1–Barnase 8×A–Venus	This paper	N/A
mTFP1–Barnase 3S–Venus	This paper	N/A
mTFP1–Barnase 7S–Venus	This paper	N/A
mTFP1–Barnase FY–Venus	This paper	N/A
mTFP1–Barnase 4Y–Venus	This paper	N/A
pcDNA5/FRT/TO V5 DNAJB1	Hageman and Kampinga, 2009	N/A
pcDNA5/FRT/TO V5 HSPA1A	Hageman and Kampinga, 2009	N/A
pwGW1/Httex1-72Q–mCherry	Arrasate et al., 2004	N/A
peGFP N1 SOD1 A4V	Polling et al., 2014	N/A
Software and algorithms		
FiJi	Schindelin et al., 2012	https://imagej.net/software/fiji/
CAMPARI	Vitalis and Pappu, 2009	http://campari.sourceforge.net
SOURSOP, release 0.1.2	Online, downloadable resource developed by the laboratories of Rohit Pappu and Alex Holehouse (no publication to cite only URL)	https://github.com/holehouse-lab/soursop
Metaboanalyst	Pang et al., 2021	https://www.metaboanalyst.ca/
Cellpose	Stringer et al., 2021	https://www.cellpose.org/
Napari	Tyson et al., 2021	https://napari.org/
MaxQuant	Cox and Mann, 2008	https://www.maxquant.org/
Other		
Resource website for this paper. Includes all the code, which is uploaded on both github and zenodo.	This paper	Github data: https://github.com/kierstenruff/RUFF_CHOI_unfolded_protein_phase_separation

CellPress
OPEN ACCESS

RESOURCE AVAILABILITY

Lead contact

Further information and requests for resources and reagents should be directed to and will be fulfilled by the lead contact, Danny M. Hatters (dhatters@unimelb.edu.au).

Materials availability

This study did not generate new unique reagents.

Data and code availability

- Raw proteomics data have been deposited PRIDE and are publicly available as of the date of publication. Accession numbers are listed in the key resources table. Additional data necessary for reproducing the figures in this manuscript, including pixels extracted from the confocal fluorescence micrographs and simulation trajectories, have been deposited at Zenodo and are publicly available as of the date of publication. The DOI is listed in the key resources table. Raw experimental images have been deposited at Mendeley and are publicly available as of the date of publication. The DOI is listed in the key resources table. Any remaining data reported in this paper will be shared by the lead contact upon request.
- All original code has been deposited at Zenodo and GitHub and is publicly available as of the date of publication. DOIs are listed in the key resources table.
- Any additional information required to reanalyze the data reported in this paper is available from the lead contact upon request.

EXPERIMENTAL MODEL AND SUBJECT DETAILS

Cell lines

Mouse Neuro2a and human HEK293T cells were used in this study. Neuro2a and HEK293T cells were maintained in opti-MEM and Dulbecco's Modified Eagle Medium (DMEM) respectively, supplemented with 10% v/v foetal bovine serum and 2 mM L-glutamine (Thermo Fisher Scientific) in a humidified incubator at 37 °C and 5% v/v atmospheric CO_2.

METHOD DETAILS

Cell imaging

For all imaging experiments, cells were plated at 3×10^4 cells per well in 8-well μ-slides (Ibidi) and transfected using Lipofectamine 3000 (Thermo Fisher Scientific) as per the manufacturer's protocol. In the case of HEK293T cells, plates were pre-coated with poly-L-lysine to aid adhesion. Imaging was conducted on a Leica TCS SP5 Confocal microscope using a HCX APO CS 63 × 1.40 Oil objective lens unless stated otherwise.

For optoDroplet experiments, cells were stained 24 h post-transfection, with Hoechst 33342 at 20 μM for 20 min at 37 °C, washed and imaged in Hank's Balanced Salt Solution (HBSS). mCherry fluorescence was imaged (561 nm excitation, 600-650 nm emission) prior to optoDroplet activation, followed by photoactivation with the 488 nm laser for 60 s at a laser intensity of 30%. mCherry and Hoechst fluorescence (excitation 405 nm, emission 420-540 nm) were then imaged immediately after activation. Droplet disassembly was observed post-activation by time-lapse imaging of mCherry fluorescence every 60 s for 15 min.

For VER-155008 treatment with optoDroplet expression experiments, Neuro2A cells were transiently transfected with barnase L14A in the optoDroplet construct for 24 h. After transfection, transfection media was removed and cells were incubated with opti-MEM containing 0, 1, 5, 10 μM VER-155008 dissolved in dimethyl sulfoxide (DMSO) for 2 or 4 h. After treatment, drug-treatment media was removed, and cells were washed twice with PBS before being stained with Hoechst 33342 and imaged on the confocal in Hank's Balanced Salt Solution (HBSS) and imaged on the confocal. Imaging for VER-155008-treated cells were conducted on a Zeiss LSM900 confocal microscope using a Plan-Apochromat 40 × 1.2 oil objective lens. With the exception of optoDroplet activation at 20% laser intensity, imaging parameters were kept the same as described above.

For chaperone optoDroplet experiments, Neuro2A cells coexpressed either opto-barnase with HSPA1A and DNAJB1, opto-barnase with HSPA1A or DNAJB1 and emerald (Y66L) or opto-barnase with emerald (Y66L). The cells expressing three constructs were transfected at a concentration ratio of 1:1:1 and cells expressing the opto-barnase with emerald (Y66L) were transfected at a 1:2 ratio. Emerald (Y66L) was used as an inert control protein to ensure the same amount of opto-barnase DNA was being added to the cells while maintaining the recommended DNA amount for lipofectamine transfection. Imaging was carried out as described above for optoDroplet experiments.

For immunofluorescence, cells were fixed 24 h post-transfection in 4% w/v paraformaldehyde for 15 min at room temperature. Cells were then permeabilized with 0.5% v/v Triton X-100 in phosphate buffered saline (PBS) for 20 mins at room temperature. Samples were blocked in 5% w/v bovine serum albumin in PBS for 1 hour at room temperature followed by staining with anti-V5 antibody (1:250 dilution, Abcam cat# ab27671) or anti-HSPA1A (1:100 dilution, Abcam cat#ab5439) diluted in PBS containing 1% w/v bovine serum albumin and 0.3% v/v Triton X-100 overnight at 4°C. Samples were then incubated in goat anti-mouse Cyanine5 (1:500) (Life

technologies cat# A10524) diluted in PBS for 30 mins at room temperature. Finally, cell nuclei were stained with Hoechst 33342 at 20 μM for 20 min at 37 °C. Cyanine5 fluorescence was imaged using 633 nm excitation and 695-765 nm emission and Hoechst using 405 nm excitation and 410-450 nm emission.

Constructs

The sequence for the DDX4-mCherry-Cry2 optoDroplet construct, which was based on the work of Shin et al. (2017), was synthesised (Thermo Fisher Scientific) and cloned into the pTriEx4 expression vector by restriction cloning using BamHI and XhoI restriction enzymes. Barnase optoDroplet constructs were generated by PCR amplification, restriction digestion using BamHI and SacI restriction enzymes, and ligation to replace the DDX4 with barnase variants. Barnase sticker variants were synthesised (Genscript) in the pTriEx4 optoDroplet expression vector. Additional barnase variants were synthesized as cassettes (GenScript) and cloned into the pTriEx4 optoDroplet expression vector using BamHI and SacI restriction enzymes. Barnase and SOD1 were cloned into the pTriEx4 FRET vectors using the FastCloning strategy (Li et al., 2011) where the inserts and vector were PCR amplified with overlapping primers, template plasmids were digested with the methylation-sensitive restriction enzyme DpnI, and the product was directly transformed in chemically competent DH5α cells. Hsp40 and Hsp70 constructs were prepared as described previously (Ormsby et al., 2013). V5-tagged chaperone proteins were overexpressed from pcDNA5/FRT/TO V5 DNAJB1 and pcDNA5/FRT/TO V5 HSPA1A provided as gifts from Harm Kampinga (Hageman and Kampinga, 2009) via Addgene. Httex1-72Q fused to mCherry in the pGW1 vector were prepared as previously described (Arrasate et al., 2004) and kindly provided by Steven Finkbeiner. All constructs were verified by sequencing.

Image analysis

Representative confocal micrographs including cell outlines were manually produced using FiJi (Schindelin et al., 2012). The brightness and contrast of individual images were adjusted to maximise the visible range of fluorescence intensity across constructs with different ranges of expression. Additional quantitative analyses on unmodified images were carried out using custom scripts written in the python programming language. Cells and nuclei were first automatically segmented using the Cellpose package (Stringer et al., 2021), and segmentation was manually inspected for quality control using Napari (Tyson et al., 2021). Cells on the image boundary, those that did not contain a nucleus, or those that were associated with more than one nucleus, were removed from subsequent analyses. Pixel coordinates were then extracted for the individual whole-cell and nuclei segmentation masks. Coordinates of nuclei were excluded from whole-cell coordinates to yield cytoplasmic pixels. For immunofluorescence experiments, compartment fluorescence was calculated as the mean intensity of pixels in the nucleus or cytoplasm respectively. Pixel intensities for individual cells were saved as csv files for further analysis as indicated below.

Extraction of c_{sat} values for optoDroplet formation of barnase

Using raw pixel data extracted from the confocal fluorescence micrographs, pixel intensities of all cells were first converted to natural log space. Cells in which greater than 25% of the pixels have the max intensity were then removed. For the remaining cells, pixel intensity histograms were generated using the data obtained prior to activation to identify the dominant peak. Since cells should have approximately uniform intensity before activation, the histograms were fit to a Gaussian distribution to filter out pixels whose intensities were not numerically similar to the mean intensity. Specifically, the Gaussian fit was used to identify the maximum frequency of the histogram and the mean intensity. The width of the distribution was then determined by finding the first instances of 20% of the maximum frequency on either side of the mean intensity. All pixels that did not fall within the intensity bins bound by this filter were removed. We also removed all pixels that were already at the maximum intensity before activation. This filtering process accounts for the fact that before activation cells should have relatively uniform intensities. The positions of the filtered pixels were then used to extract the relevant pixels from the data obtained after activation. Raw intensity histograms of the before and after activation data were then created using the filtered pixels. We further removed cells in which histograms had data in less than or equal to five bins and had fewer than 100-pixel positions. These filters ensured there were enough data for a Gaussian fit of the histograms to be reasonable. Then, the before activation histogram was fit to a single Gaussian and the mean intensity before activation ($I_{dil,before}$) was collected. This mean intensity should be proportional to the total concentration of barnase. The after-activation histogram was then fit to a model that is a mixture of two Gaussians. The premise was that if phase separation occurred there would be low intensity and high intensity peaks, where the mean of the low intensity peak ($I_{dil,after}$) is proportional to the concentration of barnase in the dilute phase and the mean of the high intensity peak ($I_{den,after}$) is proportional to the concentration of barnase in the dense phase. We restricted the fit such that the $I_{dil,after} \leq I_{dil,before}$, since the concentration of barnase in the dilute phase should not be greater than the total barnase concentration. Any cells in which $I_{dil,after}$ or $I_{dil,before}$ were less than zero or the R^2 value for the after-activation fit was less than 0.85 were removed. For the remaining cells, $I_{dil,after}$ was then collected.

Next, if there was no phase separation $I_{dil,before}$ and $I_{dil,after}$ would follow a one-to-one correspondence, i.e., $I_{dil,before} \approx I_{dil,after}$. It follows that the c_{sat} for phase separation should correspond to the intensity at which this one-to-one correspondence no longer holds. To extract the c_{sat}, we first removed outliers in the $I_{dil,before}$ versus $I_{dil,after}$ plots using three steps. Before any step was performed two threshold values were set. The value x_{1to1} = 1000 corresponded to $I_{dil,before}$ - $I_{dil,after}$ threshold for a cell to still be considered within the one-to-one regime. Then, for all cells outside of this regime, m=0.9*mean($I_{dil,before}$ - $I_{dil,after}$) was calculated. Here, m was used to distinguish between cells slightly outside the one-to-one regime or largely outside this regime. The first step to remove

CellPress
OPEN ACCESS

outliers consisted of removing cells that fell off the diagonal even though other cells in this intensity regime showed one-to-one behaviour. Next additional outliers were removed using Cook's distance (Cook, 1977). Specifically, any cells that corresponded to $I_{\text{dil,before}}$ - $I_{\text{dil,after}} < m$ were fit using a linear regression model and any cells that were 5*mean(Cook's distance for all points) were filtered out. Finally, cells well outside the one-to-one regime ($I_{\text{dil,before}}$ - $I_{\text{dil,after}} \geq m$) were fit using a linear regression model and any cells that were 5*mean(Cook's distance for all points) were filtered out.

Once outliers were removed, each barnase variant was checked for whether it had at least three off-diagonal cells ($I_{\text{dil,before}}$ - $I_{\text{dil,after}} \geq m$) so a fit for c_{sat} could be performed. Barnase variants that did not satisfy this cut-off were defined as not undergoing phase separation. For the remaining barnase variants, 50 bootstrapping trials were performed with the sample number corresponding to 0.9 times the number of cells corresponding to $I_{\text{dil,before}}$ - $I_{\text{dil,after}} \geq m$. Then these cells and the cells corresponding to the one-to-one regime were systematically split into two data sets to extract the optimal fit of c_{sat}. Specifically, all cells corresponding to $I_{\text{dil,before}}$ $\geq$ splitVal were fit using a linear regression that crossed the one-to-one line at *splitVal*. The *splitVal* that minimized both the sum of squares due to error and 1-R^2 was taken to be the c_{sat} for phase separation. The source code can be accessed via Zenodo (https://doi.org/10.5281/zenodo.6617308).

Fitting of c_{sat} versus ΔG°_U

The fraction of unfolded proteins for a given ΔG°_U of unfolding is given by:

$$p_U = \frac{e^{-\Delta G^\circ_U/RT}}{1+e^{-\Delta G^\circ_U/RT}}$$

where ΔG°_U is the standard state free energy of unfolding, R is the gas constant (8.131 J/mol-K) and T is the temperature (293 K). However, even variants with ΔG°_U = 13000 J/mol, which equates to >99% folded molecules, were able to undergo phase separation. This suggested barnase variants are more unstable in cells than their *in vitro* ΔG°_U values implied. Thus, we defined the fraction of unfolded proteins for the shifted ΔG°_U as follows:

$$p_U = \frac{e^{-\left(\Delta G^\circ_U + \Delta G^\circ_S\right)/RT}}{1+e^{-\left(\Delta G^\circ_U + \Delta G^\circ_S\right)/RT}}$$

where ΔG°_S denotes the constant offset. We then fit the extracted c_{sat} values assuming phase separation occurs at a critical unfolded concentration, using $c^* = c_{\text{sat}} \times p_U$.

c^* confidence interval

To identify hydrophobic and hydrophilic blobs in the barnase sequences we utilized the method of Lohia et al. (2019). Briefly, the average scaled Kyte-Doolittle hydrophobicity score (0 to 1) was calculated over three residue windows. Four or more contiguous windows with a score > 0.37 was considered a hydrophobic blob, whereas four or more contiguous windows with a score of $\leq$ 0.37 was considered a hydrophilic blob. We defined the change in blobs from wild type as the sum of the magnitude of the decrease in size of hydrophobic blobs and the increase in size of hydrophilic blobs. To determine the confidence interval for c^* a picking weight for each barnase variant sequence was determined by (10-(|decrease in size of hydrophobic blobs| + increase in size of hydrophilic blobs))/10, to ensure that sequences that had limited change in blobs were picked more often. Then 1000 bootstrapping trials were performed selecting 10 sequences each time based on these weights. Each trial was fit as described above to extract ΔG°_S and c^*. Then, the mean and standard deviations of these values were calculated. The interval corresponds to plotting $c_{\text{sat}} = c^*/ p_U$ with (mean(c^*)+std(c^*), mean(ΔG°_S)-std(ΔG°_S)) and (mean(c^*)-std(c^*), mean(ΔG°_S)+std(ΔG°_S)).

Computational mutagenesis study predicting ΔG°_U

To assess the effect of mutations on the stability of barnase, the x-ray structure of barnase (PDB ID: 1A2P, resolution of 1.5 Å) was obtained from the Protein Data Bank. We further processed the 3D structure by removing redundant chains, ions, water molecules and alternative conformations of residues 28, 31, 38, 85 and 96. The stability changes upon mutations, measured as the change in Gibbs Free Energy ($\Delta\Delta G$ in kcal/mol), were predicted using "Calculate Mutation Energy (Stability)" in Discovery Studio 2018 (https://www.3ds.com/products-services/biovia/products/molecular-modeling-simulation/biovia-discovery-studio/) with preliminary minimization of wild-type structure. The results of single and double mutations were used to build a transformation model to adjust the predicted $\Delta\Delta G$ of high multiple mutations (up to 8 mutations per case).

Atomistic simulations

Atomistic simulations were performed using the ABSINTH implicit solvation model and forcefield paradigm (Vitalis and Pappu, 2009) as implemented in the CAMPARI simulation engine (http://campari.sourceforge.net). Simulations were performed using a parameter set based on abs3.2_opls.prm. Parameter files, key files, and simulation trajectories can be downloaded from Zenodo (https://doi.org/10.5281/zenodo.6603909). Each simulation was performed in a spherical droplet of radius 150 Å (barnase, SOD1) or 200 Å (HSPB1, GSTP1, PRDX1, RPS28, H3-3A) at 335 K. The droplet radius was increased for the additional IFPs given that the sequence length of many of these IFPs is ~200. Additionally, counterions and an excess of 5 mM NaCl were modelled explicitly. Each

Metropolis Monte Carlo simulation comprised 10^7 equilibration steps and 5.15×10^7 production steps. For each construct, we performed five independent simulations. To model the unfolded state, simulations were started from completely random structures. For Httex1-49Q, we reanalyzed simulations performed at 335 K from the work of Warner et al. (2017). Three independent reference Flory Random Coil (FRC) simulations were performed for each construct as described in Holehouse et al. (2015). Briefly, backbone and side-chain dihedral angles were randomly drawn from previously generated dipeptide simulations to construct ensembles in which chain-chain and chain-solvent interactions were counterbalanced.

Identifying stickers from atomistic simulations of unfolded states

The mean contact probability for each residue was calculated using the SOURSOP analysis package https://github.com/holehouse-lab/soursop. Here, the probability that a residue is in contact with another residue is averaged over all residues, excluding the nearest and second nearest neighbor contacts. The cut-off for a contact was set to 5 Å. Given that the mean contact probability will be dependent on both sequence length and amino acid sequence, we also calculated the mean contact probability for each construct from the reference FRC simulations. Strong stickers should prefer chain-chain interactions and thus have a larger mean contact probability than what is observed in the corresponding FRC simulation in which chain-chain and chain-solvent interactions are counterbalanced. Therefore, we defined a strong sticker by a mean contact probability greater than the maximum mean contact probability from the corresponding FRC simulation.

To identify the type(s) of residues as most likely stickers we grouped residues into six categories. The aliphatic residues included Ala, Ile, Leu, Met, and Val; the aromatic residues included Phe, Trp, and Tyr; the unique residues included Cys and Pro; the acidic residues included Asp and Glu; the basic residues included His, Lys, and Arg; finally, the polar residues included Gly, Asn, Gln, Ser, and Thr. We calculated the fraction of total mean contact probability for each type and compared it to the expected total mean contact probability based on the number of residues in the sequence of that given type. Residue types featuring a high fraction of mean contact probability that is also greater than what we expect based on their numbers of occurrence within the sequence were defined as the predominant stickers.

Flow cytometry

HEK293T cells were plated on a poly-L-lysine-coated 24-well plate (Falcon) at a density of 7.5×10^4 cells per well and transfected with lipofectamine 3000 as per manufacturer's protocol. Following 48 h after transfection, HEK293T cells were washed once with PBS and detached by gentle pipetting and transferred into a U-bottom microplate. Flow cytometry was performed as described previously (Wood et al., 2018). Flow cytometry data were processed with FlowJo (Tree Star Inc.) to exclude un-transfected cells and cell debris and compensate the Venus channel to remove bleed-through from the mTFP1 and FRET channels. The mTFP1, Venus and FRET data were exported as csv files for further analysis. Barnase A_{50} were calculated as previously described (Wood et al., 2018).

LC-MS/MS sample preparation and analysis

Sample preparation for proteomics

Four biological replicates were used for each sample group. 1.7×10^6 HEK293T cells were seeded in 25 cm^2 flasks 24 h before transfection. Cells were transfected with barnase variants in the FRET construct. Cells were transiently transfected as per manufacturer's protocol, the transfection media was removed 6 h post-transfection and cells were incubated for a total of 48 h, including the 6 h incubation in the transfection media. Post-transfection, cells were washed and harvested in PBS by gentle pipetting and incubated in 200 μg/ml digitonin dissolved in PBS for 20 min at room temperature to remove diffuse cytosolic proteins. The cell solution was pelleted, the supernatant was collected as the soluble fraction and the pellet was resuspended with RIPA lysis buffer (150 mM NaCl, 50 mM Tris-HCl, pH 8.0, 1% NP-40, 0.5% sodium deoxycholate, 0.1% SDS, Complete EDTA-free protease inhibitor (Roche), 25 U/ml benzonase) and incubated for 10 min at room temperature. The solution was vortexed and pipetted up and down several times. 8 M urea dissolved in 50 mM Tris-HCl (pH 8.0) was added to the solution to a final concentration of 4 M and incubated for 15 min at room temperature and sonicated for 15 min. The solution was pelleted by centrifugation (21000 ×g; 15 min; 4 °C), and the supernatant was collected. The protein concentration was determined using a bicinchoninic acid assay (BCA), as per the manufacturer's protocol (Thermofisher) and 100 μg of each sample was incubated in ice-cold acetone overnight at –20°C. The acetone-precipitated samples were pelleted by centrifugation (20000 ×g; 30 min; 4°C), the acetone was removed, and the pellets were dried until all acetone had evaporated. The pellets were resuspended and incubated in 50 mM TEAB, 8 M urea (pH 8.0) for 30 min at 37°C. Proteins were reduced with tris(2-carboxyethyl)phosphine (TCEP) added to a final concentration of 10 mM and incubation for 45 min at 37°C. Proteins were alkylated with iodoacetamide added to a concentration of 55 mM, and incubation for 45 min at 37°C. The samples were diluted in TEAB to a final concentration of 1 M urea and digested overnight at 37°C with 2.5 μg of trypsin. Neat formic acid was added to a final concentration of 1% (v/v) and a sample cleanup using a solid phase extraction (SPE) method was performed. SPE cartridges (Waters/Oasis) were first equilibrated with 80% acetonitrile, 0.1% trifluoroacetic acid (TFA) followed by 0.1% TFA prior to loading samples onto the column. Bound peptides were washed twice with 1.5 ml 0.1% TFA and then eluted in 800 μl 80% acetonitrile, 0.1% TFA. Peptide samples were vacuum dried with a SpeedVac vacuum concentrator and resuspended in double-distilled water for tandem mass tag (TMT) labelling.

TMT labelling for proteomics

TMT labelling was conducted based on the manufacturer's protocol (Thermofisher). 1 M TEAB and acetonitrile were added to each sample to a concentration of 30% acetonitrile and TMT labelling reagents were resuspended in acetonitrile. TMT label reagents were added to samples in an 8:1 mass ratio and incubated for 1 at room temperature h. Samples were mixed by vortexing at regular intervals during incubation. The reaction was quenched with 8 μl of 5% hydroxylamine for 15 min at room temperature. Each label was combined in a 1:1 mass ratio for MS analysis.

Mass spectrometry data acquisition and analysis

10 μg of TMT-labelled peptide mixtures were lypophilised using a SpeedVac vacuum concentrator and resuspended to a final concentration of 0.5 μg/μl in 2% (v/v) acetonitrile, 0.05% (v/v) TFA. Peptides were analysed by nanoESI-LC-MS/MS using the Thermo Orbitrap Q Exactive Plus mass spectrometer (Thermofisher) equipped with a nanoflow reversed-phase-HPLC (Ultimate 3000 RSLC, Dionex) fitted with an Acclaim Pepmap nano-trap column (Dionex—C18, 100 Å, 75 μm× 2 cm) and an Acclaim Pepmap RSLC analytical column (Dionex—C18, 100 Å, 75 μm× 50 cm) by the University of Melbourne Mass Spectrometry and Proteomics facility. 0.6 μg of the TMT-labelled peptide mixture was loaded onto the enrichment (trap) column at an isocratic flow of 5 μl/min of 2% acetonitrile containing 0.1% (v/v) formic acid for 5 min. The enrichment column was then switched in-line with the analytical column. The eluents used for the liquid chromatography were 0.1% (v/v) formic acid, 5% (v/v) DMSO for solvent A and 0.1% formic acid (v/v), 5% (v/v) DMSO in acetonitrile for solvent B, flowed at 300 nl/min using a gradient of 3–22% solvent B in 90 min, 22–40% solvent B in 10 min and 40–80% solvent B in 5 min then maintained for 5 min before reequilibration for 8 min at 3% B prior to the next analysis. All spectra were acquired in positive ionization mode with full scan MS acquired from *m/z* 300-1600 in the FT mode at a mass resolving power of 120 000, after accumulating to an AGC target value of $3.0e^6$, with a maximum accumulation time of 25 ms. The RunStart EASY-IC lock internal lockmass was used. Data-dependent HCD MS/MS of charge states > 1 was performed using a 3 s scan method, isolation width of 0.7 m/z, at a normalised AGC target of 200%, automatic injection time, a normalised collision energy of 30% and with spectra acquired at a resolving power of 30000 (TurboTMT activated). Dynamic exclusion was used for 20 s.

Data analysis was conducted using MaxQuant (version 2.0.1.0.) (Cox and Mann, 2008) and database searches were conducted using the Swissprot Homo sapiens database (accessed on 6th July 2021, 20371 entries) with the additional barnase WT, 8×A, 9S, mTFP1 and Venus proteins. The search was conducted with 20 ppm MS tolerance, 0.5 Da MS/MS tolerance and 2 missed cleavages allowed. Oxidation (M) and acetyl (Protein N-term) variable modifications were allowed and a fixed modification for carbamidomethyl (C) was used for all samples. The false discovery rate was set at 1% for both peptides and proteins. Peptide and protein abundances were normalized by the total abundance of all identified proteins in each sample group. Proteins that were identified in less than or equal to three of the replicates in any of the sample groups were excluded from analysis. For proteins with missing values in one out of the four replicates, the missing value was filled with the mean abundance of the protein in the sample group. The initial data cleanup and normalization was conducted in python and the source code can be accessed via Zenodo (https://doi.org/10.5281/zenodo.6617308).

Multivariate analysis of proteomics data was conducted using the online software Metaboanalyst (Pang et al., 2021). A one-way ANOVA with a p-value cutoff of 0.05 using a Fisher's LSD posthoc test was conducted to determine the proteins that were significantly differently enriched in the UPODs of the barnase variants. Gene ontology search was conducted using PANTHER database (Mi et al., 2021). For visualization of the abundance z-scores a smoothing procedure was performed in which the mean value was scaled based on its p-value following a one-sample t-test (Cox et al., 2022).

Assignment of functional categories

Functional categories in Figure S5E were determined by mining the Gene ontology (biological process) and Gene ontology (molecular function) categories using UniProt, as well the function listed in the Human Protein Atlas for each of the top 94 differentially enriched proteins (Table S5) (The UniProt Consortium, 2020; Uhlén et al., 2015).

Sequence feature analysis

Ninety-one sequence features were examined for each of the top 94 differently enriched endogenous proteins by a one-way ANOVA in the proteomics dataset. Many of the sequence features were the same as those identified by Zarin et al., to be important for the molecular function of disordered regions (Zarin et al., 2019). We added additional sequence features that have been shown to be important for function or phase behavior of disordered regions. We focused on these features as they were likely to be important for interactions in the unfolded state as well. The sequence features were split into 2 distinct categories: (1) patterning and (2) composition. For the patterning features a modified version of NARDINI was deployed (Cohan et al., 2022). The specific modifications were as follows: we generated 10^3 scrambled sequences per variant rather than the 10^5 and each distribution was not fit to a gamma distribution, as these changes did not have large effects on the overall outcome. Additionally, we set g, the number of residues in a sliding window, to be 5 and 6 and take the mean of these results. In NARDINI, the residue groups were defined as follows: pol ≡ (S, T, N, Q, C, H), hyd ≡ (I, L, M, V), pos ≡ (K, R), neg ≡ (E, D), aro ≡ (F, W, Y), ala ≡ (A), pro ≡ (P), and gly ≡ (G). Z-scores below zero imply that the original sequence was more well-mixed with respect to the residue groups compared to the scrambled sequences. Z-scores above zero imply that the original sequence was blockier with respect to the residue groups compared to the scrambled sequences.

The composition features consisted of 55 features including: the fraction of each amino acid (20); the fraction of positive, negative, polar, aliphatic, aromatic, charged, chain expanding, disorder promoting, and (R, Y) residues (9 in all); the ratio of Rs to Ks and Es to Ds (2); the net charge per residue, the mean hydrophobicity, the isoelectric point, the polyproline-II propensity, and the number of (R, Y) residues (5). Additionally, we included patch features which were calculated as the fraction of the sequence that were made up of all patches of a particular amino acid or RG. Here, a patch was defined to have at least four occurrences of the residue or two occurrences of RG and to not extend past two interruptions. This led to an additional 19 features given that there were no M or W patches in the proteomics dataset. localCIDER was used to extract a majority of the composition sequence features (Holehouse et al., 2017). To calculate z-scores, the composition features were calculated over all 2269 mapped proteins in the proteomics dataset. Then the z-score for each of the top 94 proteins was calculated using the mean and standard deviation of 2269 proteins.

Finally, we also added an abundance feature to our analysis. Here, the mean and standard deviation of abundance in UPODs was calculated over all barnase variants and replicates for all 2269 mapped proteins in the proteomics dataset. These values were used to calculate the abundance z-score for each of the top 94 proteins. Together, this yielded 92 distinct z-scores for each of the top 94 proteins.

We extracted the set of proteins that were significantly enriched in a specific set of barnase UPODs compared to either all remaining barnase UPODs (Rest) or a different set of barnase variants using a *post hoc* Fisher's least significant difference (LSD) test following an ANOVA test. To determine which features were distinct to a set of proteins enriched in a specific barnase UPOD(s), we compared the z-score distribution of the 92 features in each enriched set to the z-score distribution of the remaining top 94 proteins using the two-sample Kolmogorov-Smirnov test. If the p-value was less than 0.05 the signed log(p-value) was recorded to identify the significant features of recruited proteins associated with a specific UPOD(s). Here, the log(p-value) was positive if the median of the z-score distribution associated with the set of proteins that are significantly enriched in a specific barnase UPOD(s) was greater than the median of the z-score distribution for the remaining top 94 proteins.

Disorder analysis

We used a previously generated in house disorder database. The database was generated using the Swissprot Homo sapiens database (accessed in May 2015, 20882 entries) (The UniProt Consortium, 2020). The predicted disorder for each sequence was determined by running MobiDB (Piovesan et al., 2021). A residue was considered disordered if the consensus prediction labeled it as disordered.

QUANTIFICATION AND STATISTICAL ANALYSIS

Statistical analyses were performed using MATLAB, Python, and Metaboanalyst. In order to quantify c_{sat}, 50 bootstrapped trials were conducted and the mean and SD were collected. For atomistic simulations, five independent replicas were performed. Four biological replicates were performed for each barnase variant in the proteomics experiments. Figure legends denote whether SD or SEM was used as a measure of dispersion. Statistical significance was determined using the method indicated in the figure legends. P-values of less than 0.05 were defined to be significant.

Molecular Cell, Volume *82*

Supplemental information

Sequence grammar underlying the unfolding and phase separation of globular proteins

Kiersten M. Ruff, Yoon Hee Choi, Dezerae Cox, Angelique R. Ormsby, Yoochan Myung, David B. Ascher, Sheena E. Radford, Rohit V. Pappu, and Danny M. Hatters

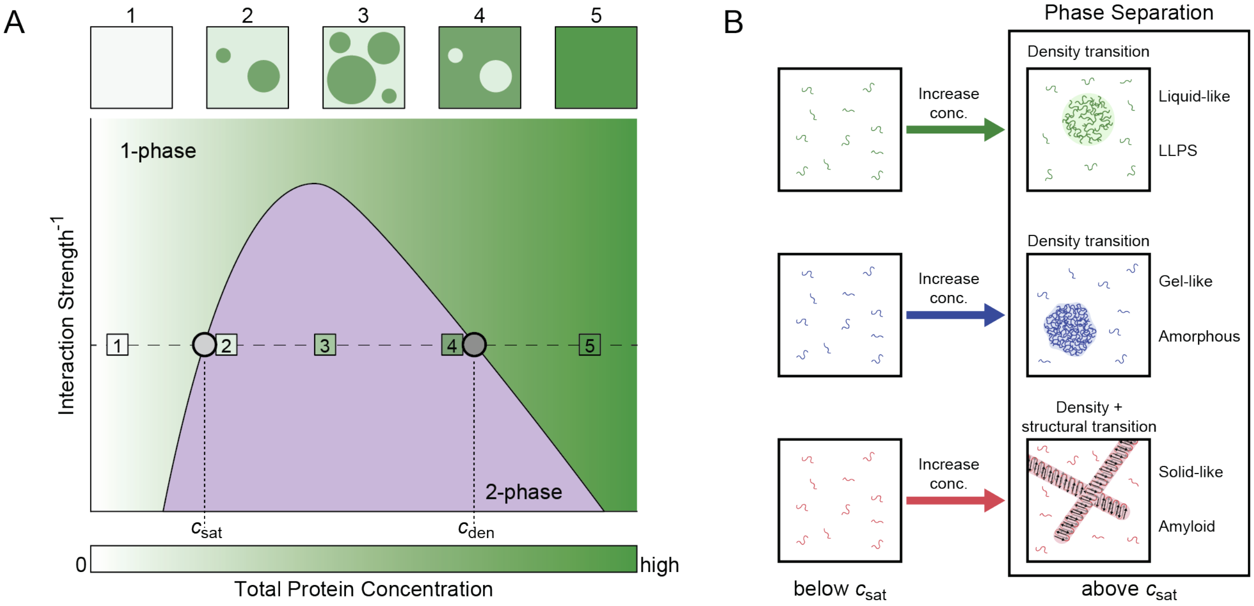

Figure S1. Phase separation is a density transition that occurs above the saturation concentration, c_{sat}. Related to Figure 1. (A) Schematic of homotypic phase separation. For a given (interaction strength)$^{-1}$ (dashed line), the system contains only one phase when the total protein concentration is less than c_{sat} (box 1). At a total protein concentration equal to c_{sat}, the system separates into a protein-deficient phase (dilute phase) and a protein-rich phase (dense phase). Above c_{sat} and below c_{den}, the concentration of the protein in the dilute phase is given by c_{sat} and the concentration of the protein in the dense phase is given by c_{den}. The volume of the dense phase increases as the total concentration of protein increases (see boxes 2-4). Finally, above c_{den} the system returns to a single phase. (B) The schematic shows how crossing c_{sat} gives rise to different types of coexisting phases, distinguished by the material properties of the dense phase.

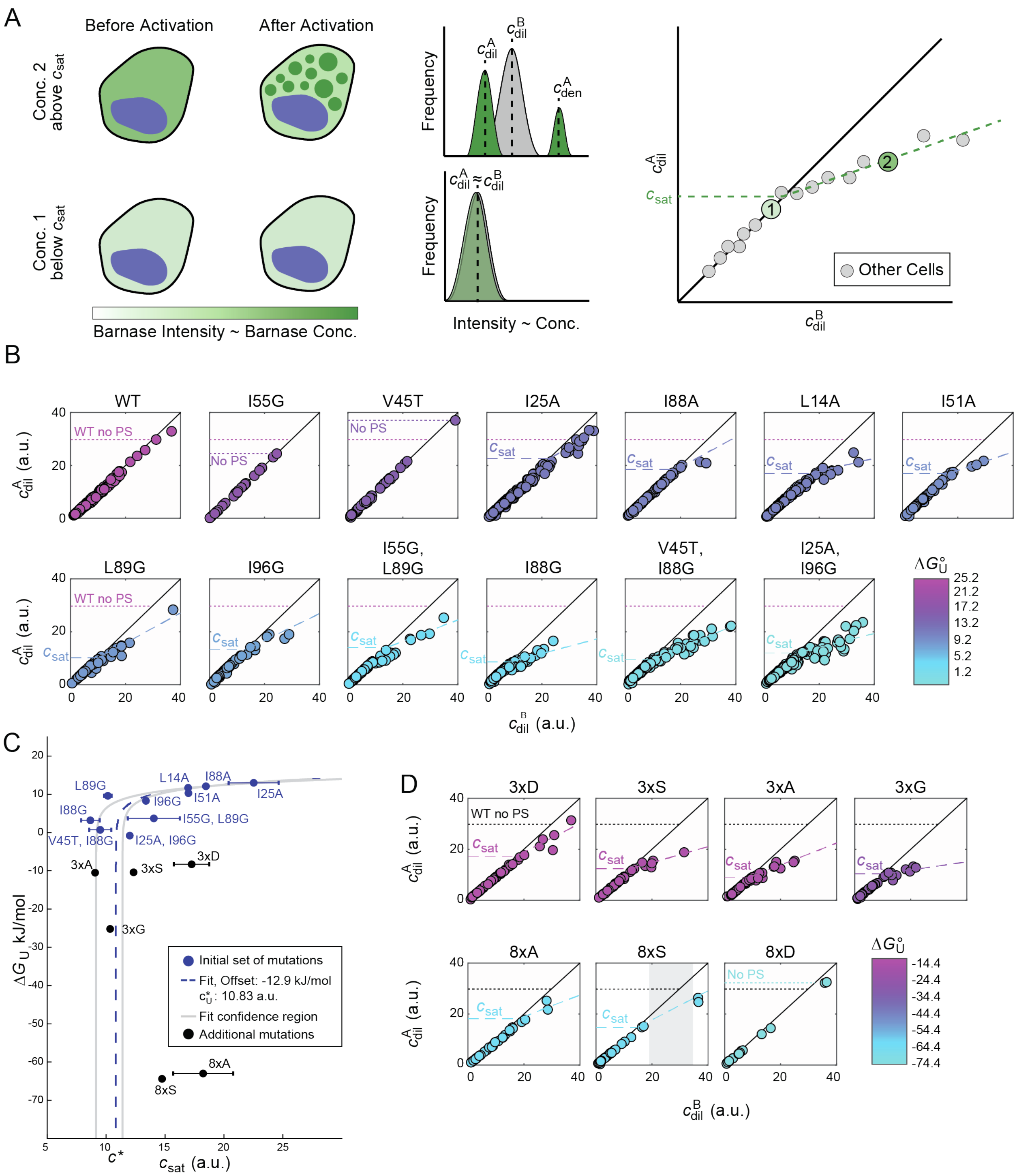

Figure S2. Extraction of c_{sat} for barnase variants. Related to Figure 3. (A) Schematic depicting how the fluorescence intensities before and after light activation can be used to extract the protein concentration (in arbitrary units) of the dilute phase before activation, c_{dil}^{B}, and the protein concentration of the dilute phase after activation, c_{dil}^{A}. Darker green colors imply higher concentrations of protein. For cells that do not undergo phase separation upon light activation, $c_{dil}^{B} \approx c_{dil}^{A}$. For cells that undergo phase separation, c_{dil}^{A} will be less than c_{dil}^{B}. Thus, the c_{sat} corresponds to where c_{dil}^{A} no longer equals c_{dil}^{B}. Blue blobs indicate the nucleus in cells. (B) c_{dil}^{B} versus c_{dil}^{A}, in arbitrary units, for the initial barnase variants examined in Figure 3. Here, each circle is a cell. Dotted lines denote that the c_{sat} must be larger than this value as no phase separation was observed up to this concentration. Dashed lines denote the fitted c_{sat} (see **STAR Methods**). (C) c_{sat} for optoDroplet formation, in arbitrary units (a.u.), versus ΔG°_{U}. Error bars indicate

standard deviations across 50 bootstrapped trials. Dashed line corresponds to a fit of the initial barnase construct data (blue circles) to a model that assumes only crossing a critical concentration of unfolded proteins is necessary for phase separation. Fit assumes that barnase is more unfolded in the cell than predicted by ΔG°_{U} = -12.9 kJ/mol with c^* = 10.83 a.u. Gray lines denote the fitted confidence interval determined by bootstrapped trials of the variants, where the variants were picked based on the degree to which they modulated the hydrophobic and hydrophilic blobs from WT (**STAR Methods**). Black points show results for additional, more extreme barnase variants in which effectively all molecules should be in the unfolded state. (D) c_{dil}^{B} versus c_{dil}^{A}, in arbitrary units, for the extreme barnase variants examined in Figure 3. Grey box in 8xS shows a void in concentrations examined which makes the exact c_{sat} value for this variant unclear.

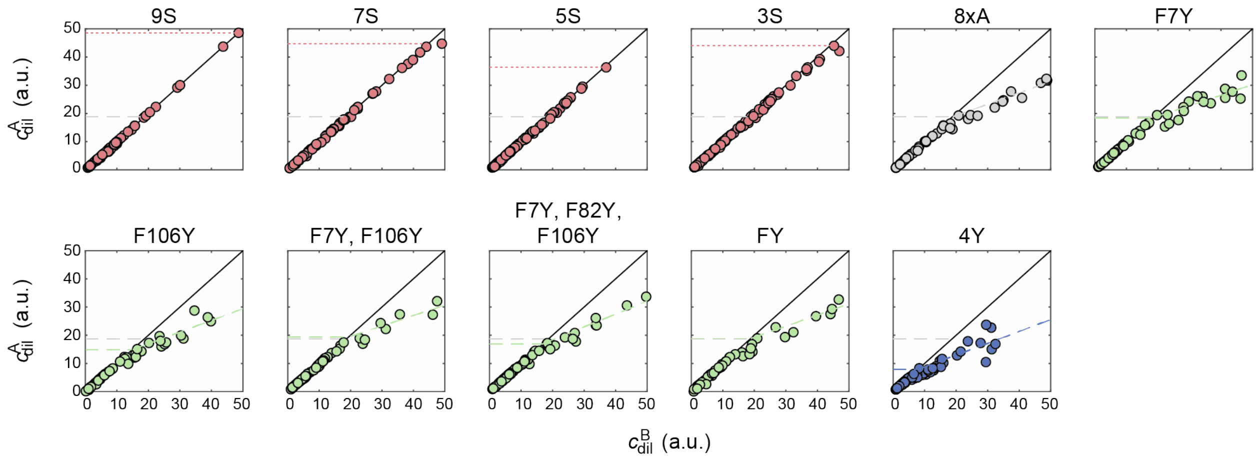

Figure S3. Extraction of c_{sat} for barnase sticker variants. Related to Figure 4. Dilute phase concentration before, c_{dil}^{B}, versus after activation, c_{dil}^{A}, in arbitrary units. Here, each circle is a cell. Dotted lines denote the c_{sat} must be larger than this value as no phase separation was observed up to this concentration. Dashed lines denote the fitted c_{sat} (see **STAR Methods**). Red points correspond to variants that reduce the number of stickers, grey to the template variant, green to variants that replace F with Y, and blue to a variant that increases the number of stickers.

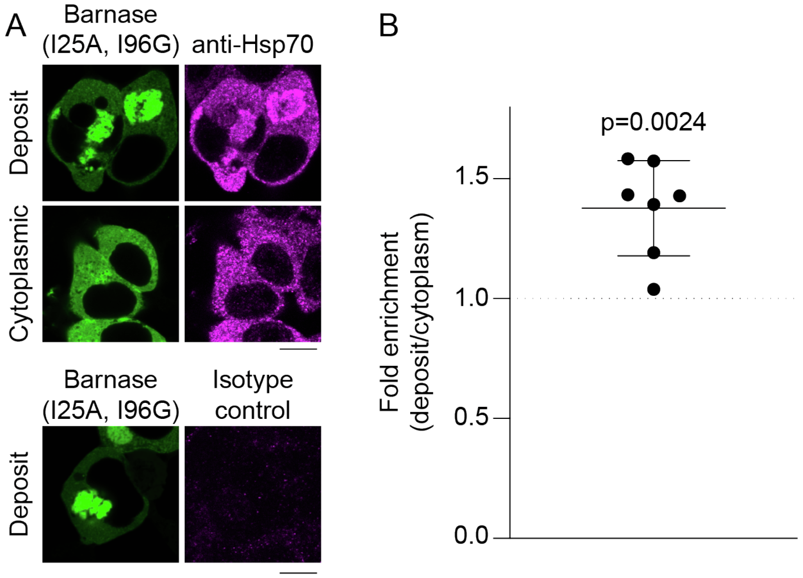

Figure S4. Hsp70 binds barnase in both the dense and dilute phase. Related to Figure 5. (A) Confocal images of Neuro2A cells transfected with the I25A, I96G barnase variant. Cells were stained by immunofluorescence for endogenous HSPA1A (Hsp70) or an isotype control as indicated. Anti-Hsp70 and isotype controls were imaged under the same conditions. Scale bar corresponds to 10 µm. (B) Graphs show quantitation of immunofluorescence. Mean and standard deviation shown, with p-value indicating results from a one-sample t-test.

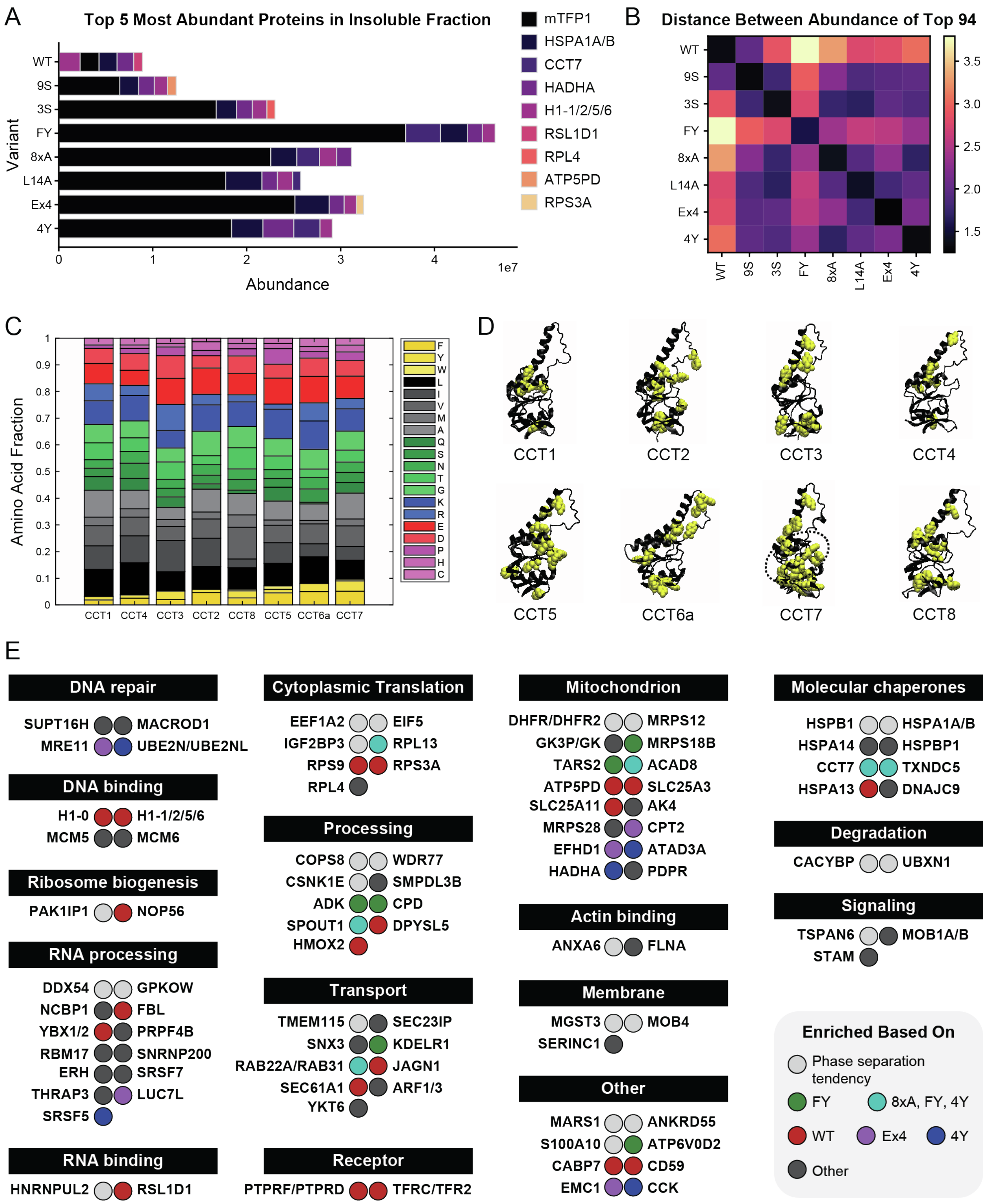

Figure S5. Barnase UPODs show selectivity not based on overlapping or synergistic biological functions. Related to Figure 6. (A) Abundance of the top 5 most abundant proteins in the insoluble fraction for each barnase variant within the set of the top 94 differently enriched endogenous proteins. Barnase (mTFP1) is the most abundant protein in each insoluble fraction, except for WT. (B) Mean Euclidean distance between $\log_{10}$(Abundance) vectors for each pair of barnase variants. Each vector includes the top 94 differently enriched endogenous proteins. Lower distances imply the compositional profiles of the insoluble fractions are more similar. (C) Amino acid fraction of the

apical domain of each of the CCT subunits. (D) AlphaFold structures of the apical domains of each CCT subunit visualized using VMD (Humphrey et al., 1996; Jumper et al., 2021; Varadi et al., 2022). Aromatic residues are shown in yellow in space filling mode. Dashed oval denotes the substrate specificity region (Joachimiak et al., 2014). (E) Top 94 differentially enriched endogenous proteins grouped by function (Shemesh et al., 2021; Uhlén et al., 2015; UniProt, 2021). The circle color denotes how the protein is enriched in the variant specific insoluble fractions, i.e., the selectivity. No clear functional groups are observed based on selectivity.
